# Family Pharmacist System for Patients With Chronic Cardiovascular or Endocrine Disease

**DOI:** 10.1001/jamanetworkopen.2025.60398

**Published:** 2026-02-23

**Authors:** Ryo Iketani, Shinobu Imai

**Affiliations:** 1 Division of Pharmacoepidemiology, Showa Medical University Graduate School of Pharmacy, Tokyo, Japan

## Abstract

**Importance:**

Japan’s family pharmacist system (FPS) has helped to prevent therapeutic duplication and drug interactions and to improve leftover drug adjustments. However, its association with lower risk of death or hospitalization remains unclear.

**Objective:**

To examine the risk of death or hospitalization from any cause among older patients with chronic cardiovascular or endocrine disease among users of an FPS.

**Design, Setting, and Participants:**

This cohort study used administrative claims data in the DeSC Health Insurance Database from April 2015 to March 2024, with a 2-year follow-up period. A prevalent new-user design was adopted to compare patients who initiated FPS use with those receiving standard pharmaceutical care. Patients 75 years of age or older with a history of visiting a pharmacy at least twice for treating hypertension, type 2 diabetes, hyperlipidemia, heart failure, angina, nonvalvular atrial fibrillation, or other arrhythmias were included.

**Exposure:**

FPS use and nonuse.

**Main Outcomes and Measures:**

The primary outcome was death or hospitalization from any cause, assessed in a time-to-first-event analysis, with each component analyzed individually as a secondary outcome. Cox proportional hazards regression with robust variance estimators was used to estimate hazard ratios (HRs) and the 95% CIs.

**Results:**

FPS users and nonusers each included 22 557 patients (total 45 114; mean [SD] age, 82.8 [5.2] years; 32 254 [71.5%] female) after time-conditional propensity score matching. The risk of death or hospitalization from any cause did not differ significantly between groups (HR, 1.00 [95% CI, 0.97-1.04]). For individual end points, the risk of death from any cause was slightly but significantly lower among users than nonusers (33.9 [95% CI, 32.0-35.9] vs 37.0 [95% CI, 35.0-39.1] deaths per 1000 person-years), with an HR of 0.91 (95% CI, 0.85-0.99). However, no significant difference in risk was observed between FPS users and nonusers for hospitalization from any cause (HR, 1.01 [95% CI, 0.98-1.05]).

**Conclusions and Relevance:**

This large-scale cohort study found that the use of an FPS was not associated with lower risk of death or hospitalization from any cause. However, the results generated a clinically important hypothesis that FPS use may be associated with slightly lower risk of death among older patients with chronic cardiovascular or endocrine disease.

## Key Points

**Question:**

Is the use of a family pharmacist system associated with lower risk of death or hospitalization among older patients with chronic cardiovascular or endocrine disease?

**Findings:**

In this cohort study including 45 114 users and nonusers of the system, there was no difference in the composite end point of death or hospitalization between users and nonusers. However, users had a slightly but significantly lower risk of death than nonusers (33.9 vs 37.0 deaths per 1000 person-years), with no significant difference in hospitalization risk.

**Meaning:**

Findings suggest that the family pharmacist system is associated with slightly fewer deaths among older patients with chronic diseases, although confirmation of a causal effect of the system on mortality will be required.

## Teaser

This cohort study assesses whether the use of the family pharmacist system in Japan is associated with the risk of death or hospitalization from any cause among older patients with chronic cardiovascular or endocrine disease.

## Introduction

The global population of older adults is projected to increase substantially by 2050.^1^ Their complicated medication regimens pose a major concern for health care professionals. As the number of comorbid chronic conditions increases, so does the number of medications used, which raises the risks of drug interactions, therapeutic duplication, and poor medication adherence, potentially leading to severe health-related problems.^2,3,4,5^ To address these challenges, the roles of pharmacists are expanding from a traditional product-oriented focus to a patient-oriented approach, facilitating collaboration with prescribers and other health care workers.^1^ Assessing the effectiveness of these expanded pharmacist roles is crucial for improving patient outcomes and guiding the appropriate allocation of medical resources.

In Japan, patients can freely choose the medical institutions and pharmacies from which they receive medications and services provided by community pharmacists, all of which are reimbursed under the national universal health care system.^6,7^ However, the concentration of pharmacies near medical institutions often leads patients to use different pharmacies depending on the institution they visit, posing a challenge to the provision of comprehensive pharmaceutical care.^8^ In April 2016, the family pharmacist system (FPS) was introduced as part of the expansion of pharmacists’ roles, with the addition of the corresponding fee (the family pharmacist consultation fee) to the medical fee schedule to facilitate centralized and consistent pharmaceutical care.^8^ This system enables patients to designate a specific community pharmacist with at least 3 years of experience, referred to as a family pharmacist, who is responsible for the patients’ pharmaceutical care. Beyond standard pharmaceutical care, family pharmacists are expected to provide expanded services, including comprehensive medication management (covering prescription drugs, over-the-counter medications, and supplements across all care settings), to share patient information with prescribers and other health care professionals through medication notebooks and clinical summaries, and to be available for consultation outside regular hours via mobile phone. Use of the FPS incurs an additional cost of approximately JPY 300 (approximately $2 US as of December 2025) per pharmacy visit beyond standard pharmaceutical care in exchange for these enhanced services. Patients who do not use the FPS continue to receive standard pharmaceutical care (hereinafter termed FPS nonusers). The FPS has been shown to improve medication adherence, facilitate the prevention of therapeutic duplication and drug interactions, and help manage leftover drugs among users.^8,9,10^

The enhanced pharmaceutical care provided by the FPS may contribute to a lower risk of severe health-related problems. A previous study reported that 10% of hospitalizations among older adults were drug-related, and two-thirds of these hospitalizations were preventable.^11^ Other studies have suggested that community pharmacists can improve laboratory values and medication adherence for patients with chronic cardiovascular or endocrine disease.^12,13,14,15,16^ Health care models similar to FPS developed in other countries have been associated with a lower risk of death, with mixed findings regarding hospitalization.^17,18^ However, evidence on whether FPS-based care is associated with lower risk of hospitalization or death remains limited. Unlike similar models elsewhere, which focus primarily on optimizing medication therapy, Japan’s FPS explicitly stipulates information sharing with multiple professionals.^8,17,18^ This structure enables broader communication among stakeholders and may hold greater potential for better outcomes. Assessing the impact of FPS is crucial to determine its value and guide resource allocation.

We hypothesized that FPS use would be associated with lower risk of hospitalization and subsequent death through enhanced pharmaceutical care. To test this hypothesis, we conducted a prevalent new-user cohort study with a nationwide health insurance database to investigate the association between FPS use and the risk of death or hospitalization among older patients with chronic cardiovascular or endocrine disease.

## Methods

Ethical review and informed consent from participants for this cohort study were waived based on the Ethical Guidelines for Medical and Biological Research Involving Human Subjects in Japan because the database used in this study was commercial and the data were deidentified. This study adhered to the Strengthening the Reporting of Observational Studies in Epidemiology (STROBE) reporting guideline for cohort studies.

### Study Design and Data Source

This prevalent new-user cohort study used data from the DeSC Health Insurance Database. The study design is shown in eFigure 1 in Supplement 1. As of January 2025, this database included 15 634 102 unique identifiers for individuals enrolled in 1 of 3 public health care insurance systems between April 2014 and March 2024. The representativeness of the entire Japanese population has been demonstrated in a previous study.^19^ This database provides claims data for diagnoses, drugs, and procedures, as well as some demographic data (eg, birth year and month and sex). Additionally, accurate death records are available for some of the database registrants.^20^ We used data from individuals enrolled in the Advanced Elderly Medical Service System, an insurance system primarily for residents aged 75 years or older, during the study period (between April 2015 and March 2024).

### Study Population

We included patients at least 75 years of age who had a history of visiting a pharmacy at least twice in the year preceding the cohort entry date for the treatment of hypertension, type 2 diabetes, hyperlipidemia, heart failure, angina, nonvalvular atrial fibrillation, or arrhythmia other than nonvalvular atrial fibrillation (eTables 1 and 2 in Supplement 1). The cohort entry date was assigned as the pharmacy visit date that met the inclusion criteria, occurring between April 2016 and March 2023. We excluded patients with a history of home medical care, dialysis, tumors, and Alzheimer disease to focus on study participants who could communicate with pharmacists themselves to primarily treat chronic cardiovascular or endocrine disease. We excluded patients with prior FPS use, outcome events on the cohort entry date, and unavailable death records. Additionally, patients receiving comprehensive claims for dispensing fees were excluded because we could not assess the claims status of dispensing fees in detail.

### Exposure

FPS users included patients who initially claimed a family pharmacist consultation fee on or after the cohort entry date, and FPS nonusers included patients who continuously claimed pharmaceutical management and instruction fees on or after the cohort entry date. Patients are usually eligible for this fee when pharmacists provide standard pharmaceutical care, including assessment, recording, and instructions on their pharmaceutical treatment. Individuals who started to claim the family pharmacist consultation fee after the cohort entry date could contribute data as users and nonusers, and we did not consider the group switch as a censor.

### Outcome Ascertainment

The primary end point was death or hospitalization from any cause, assessed in a time-to-first-event analysis. Each primary end point component was individually evaluated as a secondary end point. Similarly, we evaluated prescription changes to capture the degree of pharmaceutical intervention. Prescription changes were defined as claims for fees related to the prevention of therapeutic duplication and drug interaction and the adjustment of leftover drugs; these claims are submitted when a prescription is modified following pharmacist inquiries to prescribers based on information obtained from patient conversations, dispensing records, medication notebooks, clinical summaries, and the prescription itself, with prescriber approval. Additional secondary end points and their corresponding analyses are detailed in eTable 3 and eFigures 2 and 3 in Supplement 1.

### Statistical Analysis

We compared FPS users and nonusers of the prevalent new-user cohort design based on time-conditional propensity score (PS) matching to avoid immortal time bias.^21,22^ To estimate the time-conditional PS, we created time-based exposure sets (eFigure 4 in Supplement 1). Furthermore, we performed logistic regression, adjusting for the baseline characteristics listed in eTable 1 in Supplement 1, and stratified by time-based exposure sets. The drug comorbidity index, based on outpatient medication use in the previous year, was used to assess patient disease severity.^23^ FPS users and nonusers were matched 1:1 with the closest time-conditional PS in chronological order after checking the positivity assumption. After a nonuser was matched as a comparator, they were excluded from subsequent matching candidates.

Continuous and categorical variables were described as means (SDs) and frequencies (percentages), respectively. The balance of baseline characteristics was checked based on absolute standardized mean differences less than 0.10.^24^

To perform the intention-to-treat analysis, we followed the matched population from the assigned index date until the earliest date of database withdrawal, March 31, 2024, or 2 years (720 days) after the index date. When evaluating hospitalization and prescription changes, death was treated as a censoring event. The incidence rates of the end points were reported as the number of events per 1000 person-years. The corresponding 95% CIs were estimated using Poisson regression. Kaplan-Meier curves were used to plot the cumulative incidence of the end points for the different groups during the follow-up period. The hazard ratio (HR) and 95% CI for end points among FPS users vs nonusers were estimated using Cox proportional hazards regression with robust variance estimators. The proportional hazards assumption was assessed using log-log plots. To provide more intuitive measures, including relative risk, absolute risk difference (ARD), and number needed to treat (NNT) at 1 and 2 years, we performed a discrete-time survival analysis with inverse probability of censoring weighting under an intention-to-treat basis.^22,25^ A per-protocol analysis was supplementarily performed. Further details and results are provided in the eMethods and eTables 4 and 5 in Supplement 1.

We conducted 2 sensitivity analyses. One analysis involved changing the matching ratio of FPS users to nonusers to 1:2. The other involved redefining nonusers to include those with pharmacy visits, regardless of claims for pharmaceutical management and instruction fees.

Subgroup analyses stratified by age, sex, and primary diseases were performed. Disease subgroups were defined based on the presence of at least 1 prescription drug indicated for the corresponding condition. The matching for each subgroup was performed using time-conditional PS estimated in the main analysis.^26^ To evaluate the additive interaction between these variables and FPS use on end points, we estimated the relative excess risk due to interaction (RERI) using HRs from Cox proportional hazards regression including an interaction term and individual variables of the exposure and stratification variable.^27,28^ The corresponding 95% CI was calculated based on the delta method.^27^

Statistical analyses were performed using SAS, version 9.4 (SAS Institute Inc). The plots were generated using R version 4.3.2 (R Foundation for Statistical Computing). The 95% CIs were based on 2-sided hypothesis tests, and a 95% CI that did not include a null value was considered statistically significant.

## Results

### Patient Characteristics

From a total of 45 114 patients, 22 557 were included in each group after time-conditional PS matching (mean [SD] age, 82.8 [5.2] years; 32 254 [71.5%] female and 12 860 [28.5%] male; mean [SD] drug comorbidity index, 1.4 [1.2]) (eTable 6 and eFigure 5 in Supplement 1). The groups were well balanced across covariates, with all absolute standardized mean differences below 0.10 (eTable 6 and eFigure 6 in Supplement 1). The incidence and risk of outcomes before matching are presented in eTable 7 in Supplement 1. The mean (SD) follow-up duration was 1.3 (0.7) years in both FPS user and nonuser groups. Lost to follow-up for the primary outcome was observed in 8244 FPS users (36.5%) and 8277 nonusers (36.7%).

### Primary Outcomes

**Figure 1.**
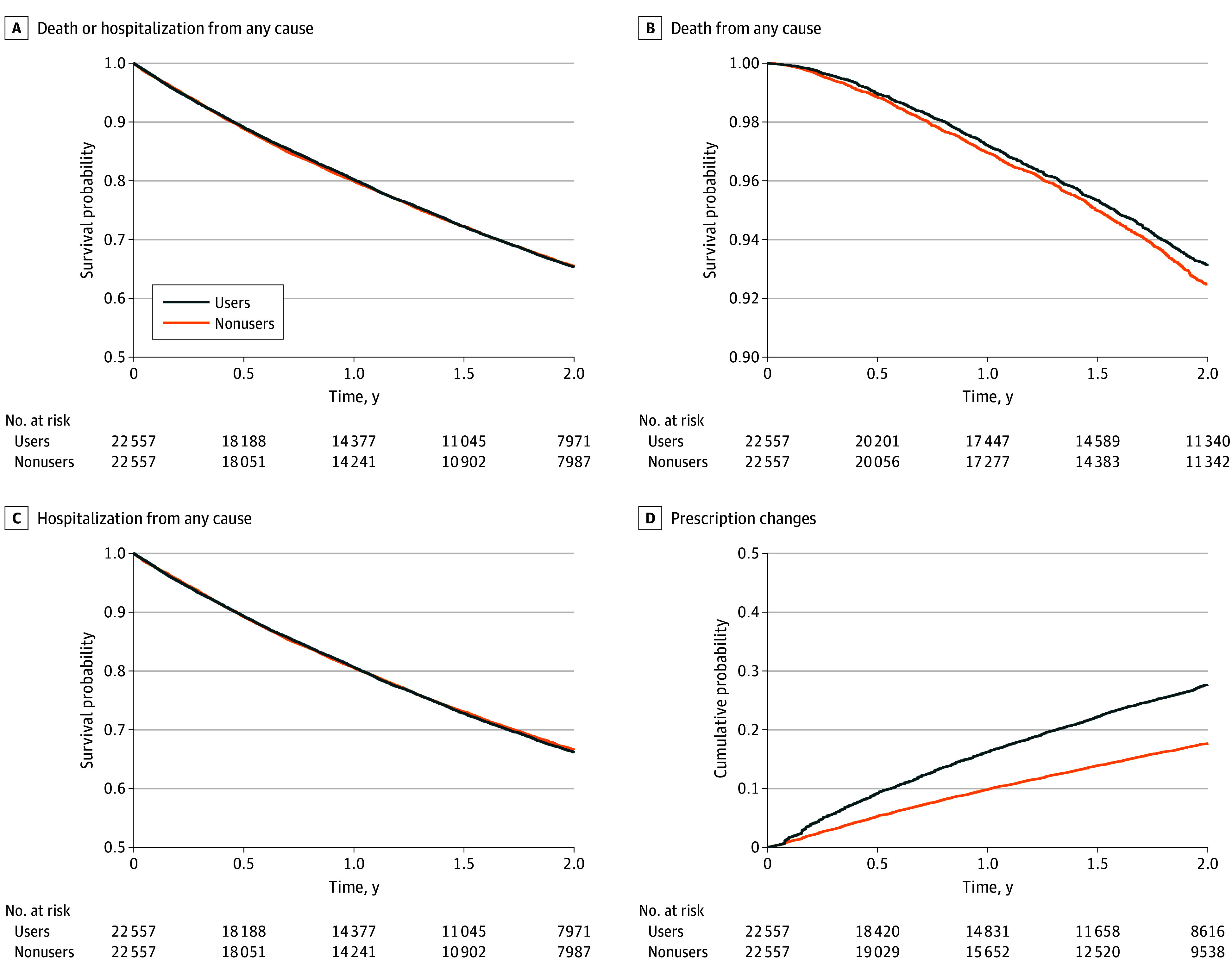
Kaplan-Meier Curves of Outcomes Comparing Family Pharmacist System Users and Nonusers After Matching

**Figure 2.**
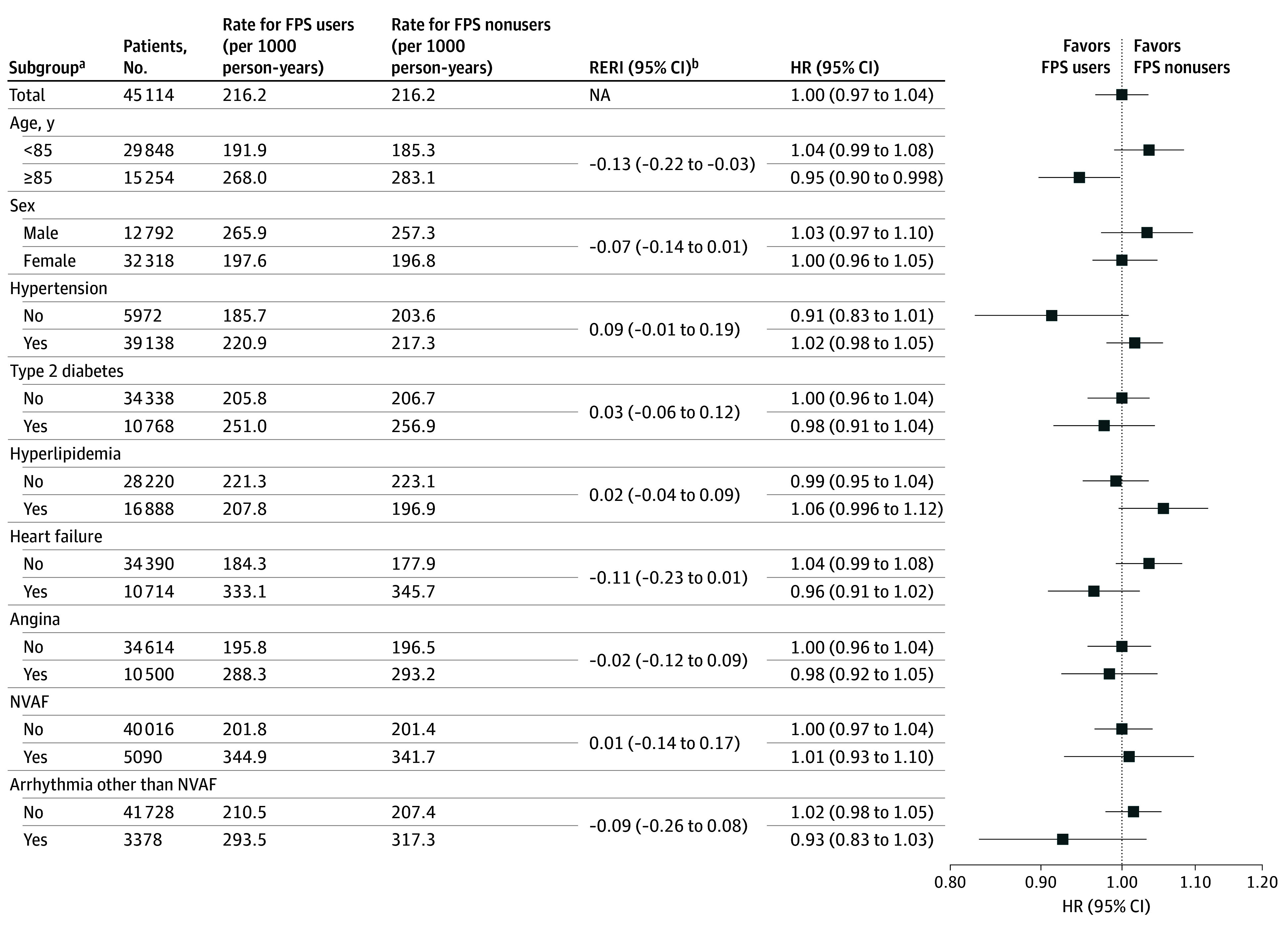
Forest Plot of Subgroup Analysis for Death or Hospitalization From Any Cause HR indicates hazard ratio; NA, not applicable; NVAF, nonvalvular atrial fibrillation. ^a^Disease subgroups were defined based on the presence of at least 1 prescription drug indicated for the corresponding condition. ^b^A positive relative excess risk due to the interaction (RERI) value indicates an antagonistic additive interaction associated with age 85 years or older, female sex, or the primary disease. Conversely, a negative RERI value suggests a synergistic additive interaction related to these factors.

In the matched population, the incidence rate of death or hospitalization from any cause was identical for both FPS users and nonusers at 216.2 (95% CI, 211.0-221.6) events per 1000 person-years (HR, 1.00 [95% CI, 0.97-1.04]) (Table and Figure 1A). Subgroup analyses revealed a significant additive interaction by age (RERI, −0.13 [95% CI, −0.22 to −0.03]) (Figure 2). Among patients aged 85 years or older, FPS users had a lower risk of the end point than nonusers (HR, 0.95 [95% CI, 0.90-0.998]).

**Table.** Incidence and Risk of Outcomes After Matching

Group	No. of patients	No. of events	Person-years	Incidence rate (95% CI), per 1000 person-years	HR (95% CI)
**Primary end point**					
Death or hospitalization from any cause					
Users	22 557	6351	29 371.1	216.2 (211.0-221.6)	1.00 (0.97-1.04)
Nonusers	22 557	6299	29 132.1	216.2 (211.0-221.6)	1 [Reference]
**Secondary end point**					
Death from any cause					
Users	22 557	1174	34 673.3	33.9 (32.0-35.9)	0.91 (0.85-0.99)
Nonusers	22 557	1271	34 367.6	37.0 (35.0-39.1)	1 [Reference]
Hospitalization from any cause					
Users	22 557	6181	29 371.1	210.4 (205.3-215.8)	1.01 (0.98-1.05)
Nonusers	22 557	6065	29 132.1	208.2 (203.0-213.5)	1 [Reference]
Prescription change					
Users	22 557	4397	30 251.1	145.4 (141.1-149.7)	1.59 (1.52-1.67)
Nonusers	22 557	2871	31 615.6	90.8 (87.5-94.2)	1 [Reference]

Abbreviation: HR, hazard ratio.

### Secondary Outcomes

In the matched population, the incidence rate of death from any cause was slightly lower for users than for nonusers (33.9 [95% CI, 32.0-35.9] vs 37.0 [95% CI, 35.0-39.1] events per 1000 person-years), with an HR of 0.91 (95% CI, 0.85-0.99) (Table and Figure 1B). Discrete-time survival analysis showed that deaths after 2 years occurred in 7.4% of users and 8.6% of nonusers based on the intention-to-treat basis (ARD, −1.2%; 95% CI, −2.1% to −0.2%; NNT, 86) (eTable 5 in Supplement 1).

The incidence rate of hospitalization from any cause was similar between users and nonusers (210.4 [95% CI, 205.3-215.8] vs 208.2 [95% CI, 203.0-213.5] events per 1000 person-years; HR, 1.01 [95% CI, 0.98-1.05]) (Table and Figure 1C). The incidence rate of prescription changes was more frequent for users than for nonusers (145.4 [95% CI, 141.1-149.7] vs 90.8 [95% CI, 87.5-94.2] events per 1000 person-years), with an HR of 1.59 (95% CI, 1.52-1.67) (Table, Figure 1D). Discrete-time survival analysis revealed that prescription changes occurred in 23.0% of users and 15.3% of nonusers after 2 years based on the intention-to-treat basis (ARD, 7.7% [95% CI, 6.7%-8.8%]; NNT, 13) (eTable 5 in Supplement 1).

Subgroup analyses of these end points are shown in eFigures 7 to 9 in Supplement 1. Subgroup analyses for death from any cause showed that FPS users were associated with lower risk of death than nonusers among female patients (HR, 0.88 [95% CI, 0.79-0.98]) and among patients with heart failure receiving pharmaceutical treatment (HR, 0.83 [95% CI, 0.74-0.94]), with significant additive interactions (eFigure 7 in Supplement 1).

### Sensitivity Analysis

The sensitivity analysis of the changing matching ratio showed results consistent with those of the main analyses across all end points (eTable 8 in Supplement 1). The sensitivity analysis that involved redefining FPS nonusers supported the main analysis results by showing greater group differences in favor of FPS users than the main analysis across all end points (eTable 9 in Supplement 1).

## Discussion

In this cohort study, FPS use was not associated with lower composite risk of death or hospitalization from any cause compared with FPS nonuse. Although subgroup analysis suggested that users aged at least 85 years had a lower risk of this end point than nonusers, the difference was marginal. Although the risk of death from any cause was slightly lower among FPS users, the risk of hospitalization was similar between groups. The discrepancy between the composite outcome of death or hospitalization and the individual risk of death can be explained by the nature of time-to-first-event analysis, which captured hospitalizations occurring prior to death. The higher frequency of prescription changes among FPS users suggests that the FPS contributes to optimized medication therapy, which may partially account for the lower risk of death, as the risk of fatal drug-related problems may be mitigated through optimizing medication therapy.^18^

Sensitivity analyses validated the robustness of our findings. The results after a change in the definition of nonusers supported the main analysis results. FPS nonusers in the sensitivity analysis included patients visiting pharmacies to receive drugs without pharmaceutical care. Variations in the risk of death and hospitalization corresponded to the degree of pharmaceutical care; however, residual confounding factors remained concerning. We could not account for social factors, such as health literacy, which may influence engagement in preventive programs and outcomes, potentially affecting the risk of death and hospitalization.^29,30^ However, if social factors functioned as confounding factors, we would also expect to have observed a higher risk of hospitalization among FPS nonusers, which was not the case.

The role of community pharmacies has contributed to desirable patient outcomes and may support lower risk of health-related problems among FPS users.^12,13,14,15,16,17,18,31,32^ A cohort study found that community pharmacy medication review was associated with a significant risk reduction of short-term death among patients after hospital discharge.^31^ Additionally, that study highlighted the risk reduction of short-term death among patients with chronic heart failure. An interaction between heart failure and FPS use, in relation to death from any cause, was observed in the present study (eFigure 7 in Supplement 1). Furthermore, FPS use was associated with a lower risk of death than was nonuse among patients with heart failure receiving pharmaceutical treatment. We evaluated the association of pharmaceutical care in patients regardless of their hospitalization history, potentially broadening the generalizability of previous findings. FPS may be particularly beneficial for managing conditions such as heart failure, which require high medication adherence, as FPS has been shown to improve adherence among users.^8,33^

Overall, several findings support a slightly but significantly lower risk of death among older patients with chronic diseases associated with FPS use. The ARD of 1.2% for death corresponded to an NNT of 86, which can be interpreted as FPS use being associated with 1 fewer death per 86 patients during 2 years. An NNT of 20 to 100 is generally considered acceptable for preventive interventions targeting serious outcomes when achievable at a low cost.^34,35^ FPS use incurs an additional cost of approximately $2 US per pharmacy visit compared with nonuse, suggesting it may be acceptable to patients at such low cost. However, the observed association with a lower risk of death is currently hypothesis-generating. Confirming a causal effect of FPS on mortality is essential, and an economic evaluation would provide further evidence regarding its cost-effectiveness. The potential burden on pharmacists also remains a consideration.^8^ Limiting FPS application to patient groups in which benefit is most supported, such as those with heart failure, may be a viable strategy for efficient allocation of medical resources.

### Strengths and Limitations

This study has strengths. We performed a prevalent new-user cohort study using a large claims database, ensuring that the results are generalizable to the broader population of FPS users in Japan and supported by high statistical power. Presenting the results for all patients as well as for individual disease subgroups provided meaningful insights for guiding the application of FPS in clinical practice.

However, our study also has limitations. First, unmeasured confounding may exist due to constraints in administrative data, particularly the absence of information on social and behavioral factors. Nevertheless, we hypothesized that FPS users would experience comparable or superior outcomes given their access to enhanced pharmaceutical services, and our results aligned with this expectation, suggesting that unmeasured confounding is unlikely to fully explain the observed associations. Second, the application of strict exclusion criteria, including removal of patients with severe conditions, limits generalizability of our results to relatively healthier subsets of older adults with cardiovascular or endocrine conditions. Subgroup analyses indicated age-related variations in the association between FPS use and health-related problems, with our primary focus on high-risk individuals aged at least 75 years. Therefore, extrapolation of these findings to younger populations should be done with caution. In addition, the use of claims data for variable definitions may have led to misclassifications. Disease subgroups defined by diagnosis codes and prescribed medications may miss some relevant patients.

## Conclusions

In this large, nationwide cohort study, FPS use was not associated with a lower risk of death or hospitalization among patients 75 years of age or older with chronic cardiovascular or endocrine disease. However, this study generated a clinically important hypothesis that FPS use was associated with slightly lower risk of death from any cause compared with FPS nonuse in this population. Confirmation of a causal effect of FPS on mortality is required.

## Notes

Supplement 1. **eMethods.** Statistical procedures for discrete-time survival analysis **eTable 1.** Definitions for variables **eTable 2.** Definitions for inclusion and exclusion criteria **eTable 3.** Incidence and risk of supportive secondary end points after matching **eTable 4.** Discrete-time survival analysis with the inverse probability of censoring weighting after matching (1 year) **eTable 5.** Discrete-time survival analysis with the inverse probability of censoring weighting after matching (2 years) **eTable 6.** Baseline characteristics before and after matching **eTable 7.** Incidence and risk of outcomes before matching **eTable 8.** Sensitivity analysis of changing the matching ratio to 1:2 (user: nonuser) **eTable 9.** Sensitivity analysis of changing the definition of nonusers **eFigure 1.** Study design diagram **eFigure 2.** Kaplan–Meier curves of death or unplanned hospitalization from any cause after matching **eFigure 3.** Kaplan–Meier curves of unplanned hospitalization from any cause after matching **eFigure 4.** Definition for time-based exposure set **eFigure 5.** Flowchart of study cohort selection **eFigure 6.** Distribution of propensity score before and after matching **eFigure 7.** Subgroup analysis for death from any cause **eFigure 8.** Subgroup analysis for hospitalization from any cause **eFigure 9.** Subgroup analysis for prescription changes

Supplement 2.
Data Sharing Statement
