# Supplementary material for: Family Pharmacist System for Patients With Chronic Cardiovascular or Endocrine Disease
Source: JAMA Netw Open. 2026 Feb 23;9(2):e2560398. doi: 10.1001/jamanetworkopen.2025.60398 (PMC12931473; doi:10.1001/jamanetworkopen.2025.60398)
Supplement: Supplement 1. — eMethods. Statistical procedures for discrete-time survival analysis eTable 1. Definitions for variables eTable 2. Definitions for inclusion and exclusion criteria eTable 3. Incidence and risk of supportive secondary end points after matching eTable 4. Discrete-time survival analysis with the inverse probability of censoring weighting after matching (1 year) eTable 5. Discrete-time survival analysis with the inverse probability of censoring weighting after matching (2 years) eTable 6. Baseline characteristics before and after matching eTable 7. Incidence and risk of outcomes before matching eTable 8. Sensitivity analysis of changing the matching ratio to 1:2 (user: nonuser) eTable 9. Sensitivity analysis of changing the definition of nonusers eFigure 1. Study design diagram eFigure 2. Kaplan–Meier curves of death or unplanned hospitalization from any cause after matching eFigure 3. Kaplan–Meier curves of unplanned hospitalization from any cause after matching eFigure 4. Definition for time-based exposure set eFigure 5. Flowchart of study cohort selection eFigure 6. Distribution of propensity score before and after matching eFigure 7. Subgroup analysis for death from any cause eFigure 8. Subgroup analysis for hospitalization from any cause eFigure 9. Subgroup analysis for prescription changes [file jamanetwopen-e2560398-s001.pdf]

Supplementary Online Content

Iketani R, Imai S. Family pharmacist system for patients with chronic cardiovascular or endocrine disease. *JAMA Netw Open*. 2026;9(2):e2560398.
doi:10.1001/jamanetworkopen.2025.60398

eMethods. Statistical procedures for discrete-time survival analysis
eTable 1. Definitions for variables
eTable 2. Definitions for inclusion and exclusion criteria
eTable 3. Incidence and risk of supportive secondary end points after matching
eTable 4. Discrete-time survival analysis with the inverse probability of censoring weighting after matching (1 year)
eTable 5. Discrete-time survival analysis with the inverse probability of censoring weighting after matching (2 years)
eTable 6. Baseline characteristics before and after matching
eTable 7. Incidence and risk of outcomes before matching
eTable 8. Sensitivity analysis of changing the matching ratio to 1:2 (user: nonuser)
eTable 9. Sensitivity analysis of changing the definition of nonusers
eFigure 1. Study design diagram
eFigure 2. Kaplan–Meier curves of death or unplanned hospitalization from any cause after matching
eFigure 3. Kaplan–Meier curves of unplanned hospitalization from any cause after matching
eFigure 4. Definition for time-based exposure set
eFigure 5. Flowchart of study cohort selection
eFigure 6. Distribution of propensity score before and after matching
eFigure 7. Subgroup analysis for death from any cause
eFigure 8. Subgroup analysis for hospitalization from any cause
eFigure 9. Subgroup analysis for prescription changes

This supplementary material has been provided by the authors to give readers additional information about their work.

© 2026 Iketani R et al. *JAMA Network Open*.

eMethods. Statistical procedures for discrete-time survival analysis

The discrete-time survival analysis was performed by dividing the 2-year follow-up period into 1-month (30-day) intervals. For the intention-to-treat analysis, we considered administrative censoring, defined as reaching March 31, 2024 or completing the follow-up period (2 years) to correct for selection bias. For the per-protocol analysis, we considered artificial censoring, defined as no claims longer than 90 days, owing to the previous claim of the family pharmacist consultation fee for users and the pharmaceutical management and instruction fee for nonusers, in addition to administrative censoring. The artificial censoring date was 90 days after the patient's last visit to the pharmacy. We also considered death as a censor when evaluating hospitalization from any cause and prescription changes. We calculated each stabilized weight for administrative censoring, artificial censoring, and death by estimating the uncensoring probability using pooled logistic regression. The numerator of the stabilized weight was estimated using months since cohort entry (linear and quadratic terms). The stabilized weight denominator was estimated using the exposure variable, time-dependent updated variables listed in eTable 1 in this supplement, and months since cohort entry (linear and quadratic terms). The final weight was calculated by multiplying the initial weight by the applied weight. Percentages, risk ratios, and absolute risk differences for outcomes were calculated based on weighted samples at 1 and 2 years after follow-up. We performed a bootstrap procedure to calculate the 95% confidence intervals of the percentage, risk ratio and absolute risk difference from 200 samples with a repetition of the inverse probability of censoring weighting. The number needed to treat was calculated when the absolute risk difference indicated a better trend in users.

© 2026 Iketani R et al. *JAMA Network Open*.

eTable 1. Definitions for variables

Variables	Definitions	Purposes
Age	Age at the index date	Inclusion criteria, covariate, subgroup analysis
Sex	Male/female	Covariate, subgroup analysis
DCI	Calculated using outpatient records within 1 year prior, based on an algorithm by Gedeborg R, et al. *Epidemiology*. 2021;32(4):607-615.	Covariate
Number of hospitalizations	The sum of non-consecutive inpatient claims	Covariate
Number of physician visits	The sum of physician consultation fee claims (procedure code[a]: A000, A001, A002, and A003)	Covariate
Number of medical facilities used	The sum of different medical facility identifiers	Covariate
Dispensing in the hospital	Dispensed in the hospital in addition to the pharmacy	Covariate
Surgery	Procedure code[a]: K	Covariate
Number of drug types taken	The sum of drugs in different chemical subgroups based on WHO-ATC codes dispensed at outpatient settings	Covariate
Home medical care	Procedure code[a]: C000, C001, C001-2, C002, C002-2, C003, C004, C004-2, C005, C005-1-2, and C005-2<br>or<br>claims code[a]: 440000810, 440001510, 440001910, 440002110, 440004610, 440005710, 440005810, 440005910, 440006510, 440006610, 440009610, 440009710, 440009810, 440010410, 440010510, 440012770, 440013170, 440013370, 440013770, 440013870, 440014010, 440014070, 440014110, 440016610, 440016710, 440018110, 440018210, 440020110, and 450001170	Exclusion criteria
Dialysis	Procedure code[a]: C102, C154, C155, C156, J038, and J042	Exclusion criteria

© 2026 Iketani R et al. *JAMA Network Open*.

eTable 1. Definitions for variables (Continued)

Variables	Definitions	Purposes
Primary disease		
Hypertension	ICD-10 code: I10, I11, I12, I13, and I15 without suspicious disease name	Covariate, inclusion criteria, subgroup analysis
Type 2 diabetes	ICD-10 code: E11 and E14 without suspicious disease name	Covariate, inclusion criteria, subgroup analysis
Hyperlipidemia	ICD-10 code: E78 without suspicious disease name	Covariate, inclusion criteria, subgroup analysis
Heart failure	ICD-10 code: I110 and I50 without suspicious disease name	Covariate, inclusion criteria, subgroup analysis
Angina	ICD-10 code: I20 without suspicious disease name	Covariate, inclusion criteria, subgroup analysis
NVAF	ICD-10 code: I48 without suspicious disease name	Covariate, inclusion criteria, subgroup analysis
Arrhythmia other than NVAF	ICD-10 code: I44, I45, I47, and I49 without suspicious disease name	Covariate, inclusion criteria, subgroup analysis
Comorbidity		
Vascular disease	ICD-10 code: I21, I22, I25, and I70 without suspicious disease name	Covariate
Stroke	ICD-10 code: I60, I61, I62, I63, and G459 without suspicious disease name	Covariate
Valvular disease	ICD-10 code: I050, I052, I080, I081, I083, I088, I340, I341, I342, I348, I350, I351, I352, I358, I359, I360, I361, I362, I369, I370, I371, I372, I379, I489, Z952, Z953, and Z954 without suspicious disease name	Covariate
Venous thromboembolism	ICD-10 code: I800, I801, I802, I803, I808, I809, and I82 without suspicious disease name	Covariate

© 2026 Iketani R et al. *JAMA Network Open*.

eTable 1. Definitions for variables (Continued)

Variables	Definitions	Purposes
Comorbidity		
Other embolisms	ICD-10 code: I26 and I74 without suspicious disease name	Covariate
Pacemaker placement	ICD-10 code: Z950 without suspicious disease name	Covariate
Gastrointestinal hemorrhage	ICD-10 code: I850, I983, K226, K250, K252, K254, K256, K260, K262, K264, K266, K270, K284, K290, K625, K661, K762, K920, K921, and K922 without suspicious disease name	Covariate
Kidney disease	ICD-10 code: N01, N03, N05, N07, N18, N19, N25, and N28 without suspicious disease name	Covariate
Liver disease	ICD-10 code: K70, K71, K72, K73, K74, K75, and K76 without suspicious disease name	Covariate
Tumor	ICD-10 code: C and D<br>or<br>WHO-ATC code: L01AA, L01AB, L01AC, L01AD, L01AX, L01BA, L01BB, L01BC, L01CA, L01CB, L01CD, L01CE, L01CX, L01DA, L01DB, L01DC, L01EA, L01EB, L01EC, L01ED, L01EE, L01EF, L01EG, L01EH, L01EJ, L01EK, L01EL, L01EN, L01EX, L01FA, L01FB, L01FC, L01FD, L01FE, L01FF, L01FG, L01FX, L01FY, L01XA, L01XB, L01XD, L01XF, L01XG, L01XH, L01XK, L01XL, L01XX, L01XY, L02AE, L02BA, L02BB, L02BG, L02BX, L03AB, L03AX, L04AA, L04AG, L04AH, L04AX, and V10XX	Exclusion criteria
Alzheimer's disease	WHO-ATC code: N06DA and N06DX	Exclusion criteria

© 2026 Iketani R et al. *JAMA Network Open*.

eTable 1. Definitions for variables (Continued)

Variables	Definitions	Purposes
Drugs		
CCB with mainly vascular effects	WHO-ATC code: C08CA01, C08CA02, C08CA04, C08CA05, C08CA07, C08CA08, C08CA10, C08CA11, C08CA12, C08CA14, C08CA15, C09DB01, C09DB04, C09DB05, C09DB07, C09DX08, and C10BX03 in oral formulation<br>or<br>drug code[a]: 2149034, 2149038, 2149043, 2149115, 2149120, 2149121, and 2149122	Covariate, inclusion criteria
CCB with direct cardiac effects	WHO-ATC code: C08DA01 and C08DB01 in oral formulation	Covariate, inclusion criteria
Other CCB	WHO-ATC code: C08EA02 in oral formulation	Covariate, inclusion criteria
ACE	WHO-ATC code: C09AA01, C09AA02, C09AA03, C09AA04, C09AA06, C09AA07, C09AA08, C09AA10, C09AA12, C09AA14, and C09AA16 in oral formulation<br>or<br>drug code[a]: 2144003	Covariate, inclusion criteria
ARB	WHO-ATC code: C09CA01, C09CA03, C09CA04, C09CA06, C09CA07, C09CA08, C09CA09, C09DA01, C09DA03, C09DA04, C09DA06, C09DA07, C09DB01, C09DB04, C09DB05, C09DB07, and C09DX08 in oral formulation<br>or<br>drug code[a]: 2149115, 2149120, 2149121, and 2149122	Covariate, inclusion criteria
ARNI	WHO-ATC code: C09DX04 in oral formulation	Covariate, inclusion criteria
Non-selective beta blocker	WHO-ATC code: C07AA01, C07AA03, C07AA05, C07AA07, C07AA12, and C07AA15 in oral formulation<br>or<br>drug code[a]: 2123006 and 2149021	Covariate, inclusion criteria

© 2026 Iketani R et al. *JAMA Network Open*.

eTable 1. Definitions for variables (Continued)

Variables	Definitions	Purposes
Drugs		
Selective beta blocker	WHO-ATC code: C07AB02, C07AB03, C07AB04, C07AB05, C07AB06, C07AB07, and C07AB08 in oral formulation	Covariate, inclusion criteria
Alpha and beta blocker	WHO-ATC code: C07AG01 and C07AG02 in oral formulation<br>or<br>drug code[a]: 2123014 and 2149018	Covariate, inclusion criteria
Loop diuretic	WHO-ATC code: C03CA01, C03CA02, C03CA03, and C03CA04 in oral formulation<br>or<br>drug code[a]: 2139008	Covariate, inclusion criteria
Thiazide	WHO-ATC code: C02LA01, C03AA03, C03AA06, C09DA01, C09DA03, C09DA04, C09DA06, C09DA07, and C09DX08 in oral formulation<br>or<br>drug code[a]: 2132006	Covariate, inclusion criteria
MRA	WHO-ATC code: C03DA01 and C03DA04 in oral formulation<br>or<br>drug code[a]: 2149049	Covariate, inclusion criteria
Other diuretics	WHO-ATC code: C03BA09, C03BA11, and S01EC01 in oral formulation<br>or<br>drug code[a]: 2149007	Covariate, inclusion criteria
Antiarrhythmic drugs	WHO-ATC code: C01BA01, C01BA02, C01BA03, C01BB02, C01BB04, C01BC03, C01BC04, C01BD01, C01BG07, and C08EA02 in oral formulation<br>or<br>drug code[a]: 2129008 and 2129012	Covariate, inclusion criteria
Fibrate	WHO-ATC code: C10AB02, C10AB05, and C10AB12 in oral formulation<br>or<br>drug code[a]: 2183007	Covariate, inclusion criteria

© 2026 Iketani R et al. *JAMA Network Open*.

eTable 1. Definitions for variables (Continued)

Variables	Definitions	Purposes
Drugs		
Statin	WHO-ATC code: C10AA01, C10AA03, C10AA04, C10AA05, C10AA07, C10AA08, C10BA05, C10BA06, and C10BX03 in oral formulation<br>or<br>drug code[a]: 2189103	Covariate, inclusion criteria
Ezetimibe	WHO-ATC code: C10AX09, C10BA05, and C10BA06 in oral formulation<br>or<br>drug code[a]: 2189103	Covariate, inclusion criteria
Other oral lipid-modifying agents	WHO-ATC code: C10AC01, C10AD01, C10AX02, and C10AX06 in oral formulation<br>or<br>drug code[a]: 2189004, 2189006, and 2189014	Covariate, inclusion criteria
Other injectable lipid-modifying agents	WHO-ATC code: C10AX13, C10AX14, and C10AX16	Covariate, inclusion criteria
Other heart failure drugs	WHO-ATC code: C01AA04, C01AA05, C01AA08, C01DX22, C01EB17, and C03XA01 in oral formulation	Covariate, inclusion criteria
VKA	WHO-ATC code: B01AA03 in oral formulation	Covariate, inclusion criteria
DOAC	WHO-ATC code: B01AE07, B01AF01, B01AF02, and B01AF03 in oral formulation	Covariate, inclusion criteria
Low-dose aspirin	WHO-ATC code: B01AC06, B01AC30, and B01AC56 in oral formulation	Covariate, inclusion criteria
Nitroglycerin	WHO-ATC code: C01DA02 in oral and external formulations	Covariate, inclusion criteria
ADP inhibitors other than ticlopidine	WHO-ATC code: B01AC04, B01AC22, and B01AC24 in oral formulation	Covariate, inclusion criteria
Ticlopidine	WHO-ATC code: B01AC05 in oral formulation	Covariate, inclusion criteria

© 2026 Iketani R et al. *JAMA Network Open*.

eTable 1. Definitions for variables (Continued)

Variables	Definitions	Purposes
Drugs		
SU	WHO-ATC code: A10BB01, A10BB02, A10BB03, A10BB09, A10BB12, A10BB31, and A10BD06 in oral formulation<br>or<br>drug code[a]: 3961002	Covariate, inclusion criteria
DPP-4 inhibitor	WHO-ATC code: A10BD09, A10BD13, A10BD19, A10BH01, A10BH02, A10BH03, A10BH04, A10BH05, and A10BH08 in oral formulation<br>or<br>drug code[a]: 3969024 and 3969025	Covariate, inclusion criteria
SGLT-2 inhibitor	WHO-ATC code: A10BD19, A10BK01, A10BK02, A10BK03, A10BK05, and A10BK07 in oral formulation<br>or<br>drug code[a]: 3969021, 3969106, and 3969107	Covariate, inclusion criteria
GLP-1 agonist	WHO-ATC code: A10AE54, A10AE56, A10BJ01, A10BJ02, A10BJ03, A10BJ05, and A10BJ06	Covariate, inclusion criteria
Insulin	WHO-ATC code: A10AB01, A10AB04, A10AB05, A10AB06, A10AC01, A10AC04, A10AD01, A10AD04, A10AD05, A10AD06, A10AE04, A10AE05, A10AE06, A10AE54, and A10AE56	Covariate, inclusion criteria
Biguanide	WHO-ATC code: A10BA02, A10BA03, A10BD05, A10BD08, and A10BD13 in oral formulation<br>or<br>drug code[a]: 3969109	Covariate, inclusion criteria
Other hypoglycemic drugs	WHO-ATC code: A10BD05, A10BD06, A10BD09, A10BF01, A10BF02, A10BF03, A10BG03, A10BX02, A10BX03, A10BX08, and A10BX15 in oral formulation<br>or<br>drug code[a]: 3969102	Covariate, inclusion criteria
Edaravone	WHO-ATC code: N07XX14	Covariate

© 2026 Iketani R et al. *JAMA Network Open*.

eTable 1. Definitions for variables (Continued)

Variables	Definitions	Purposes
Drugs		
Subarachnoid hemorrhage drugs	WHO-ATC code: C04AX32, and C04AX33<br>or<br>drug code[a]: 2190418 and 3999411	Covariate
NSAIDs	WHO-ATC code: M01AB01, M01AB02, M01AB05, M01AB08, M01AB11, M01AB14, M01AC01, M01AC05, M01AC06, M01AE01, M01AE02, M01AE03, M01AE09, M01AE11, M01AE12, M01AG01, M01AG03, M01AH01, M02AA07, M02AA08, M02AA10, M02AA11, M02AA13, M02AA15, M02AA19, M02AA23, and M02AA31 in oral and external formulations<br>or<br>drug code[a]: 1147006, 1149010, 1149019, 1149029, 1149030, 2649733, and 2649896	Covariate
Acetaminophen	WHO-ATC code: N02AJ13, and N02BE01 in oral and external formulations	Covariate
Opioid	WHO-ATC code: N01AH01, N02AA01, N02AA03, N02AA05, N02AB03, N02AJ13, N02AX02, N02AX06, and N07BC02 in oral, injectable, and external formulations<br>or<br>drug code[a]: 641140109	Covariate
Steroid	WHO-ATC code: D07AA03, D07AB01, D07AB02, D07AB09, D07AB10, D07AB19, D07AC01, D07AC04, D07AC06, D07AC07, D07AC08, D07AC10, D07AC11, D07AC13, D07AC15, D07AC19, D07AD01, D07CA01, D07CA03, D07CC01, D07CC02, D07XA01, D07XB05, H02AA02, H02AB01, H02AB02, H02AB04, H02AB06, H02AB08, H02AB09, and H02AB10 in oral and external formulations<br>or<br>drug code[a]: 2646729	Covariate

© 2026 Iketani R et al. *JAMA Network Open*.

eTable 1. Definitions for variables (Continued)

Variables	Definitions	Purposes
Drugs		
BZD	WHO-ATC code: N05BA01, N05BA02, N05BA03, N05BA05, N05BA06, N05BA08, N05BA09, N05BA12, N05BA17, N05BA18, N05BA19, N05BA21, N05BA22, N05BA23, N05BA25, N05CD01, N05CD02, N05CD03, N05CD04, N05CD05, N05CD06, N05CD09, N05CD10, and N05CD15 in oral formulation<br>or<br>drug code[a]: 1124005, 1124013, 1124024, 1124027, and 1129006	Covariate
Non-BZD	WHO-ATC code: N05CF01, N05CF02, and N05CF04 in oral formulation	Covariate
Orexin receptor blocker	WHO-ATC code: N05CJ01 and N05CJ02 in oral formulation	Covariate
H2 blocker	WHO-ATC code: A02BA01, A02BA02, A02BA03, A02BA04, A02BA06, and A02BA08 in oral formulation	Covariate
PPI	WHO-ATC code: A02BC01, A02BC03, A02BC04, A02BC05, A02BC08, A02BD03, A02BD07, A02BD12, A02BD13, A02BD14, and A02BD15, B01AC56 in oral formulation	Covariate
Antibiotic	WHO-ATC code: J01AA01, J01AA02, J01AA07, J01AA08, J01BA01, J01CA01, J01CA04, J01CA06, J01CR02, J01CR04, J01DB01, J01DB11, J01DC02, J01DC04, J01DC07, J01DD08, J01DD13, J01DD15, J01DD16, J01DD17, J01DD18, J01DI03, J01FA01, J01FA02, J01FA06, J01FA07, J01FA09, J01FA10, J01FA12, J01MA01, J01MA02, J01MA06, J01MA07, J01MA12, J01MA14, J01MA17, J01MA19, J01MA21, J01MA22, and J01MA25 in oral formulation	Covariate

© 2026 Iketani R et al. *JAMA Network Open*.

eTable 1. Definitions for variables (Continued)

Variables	Definitions	Purposes
Drugs		
Antidepressant	WHO-ATC code: N06AA02, N06AA04, N06AA06, N06AA07, N06AA09, N06AA10, N06AA16, N06AA17, N06AA21, N06AB05, N06AB06, N06AB08, N06AB10, N06AX03, N06AX05, N06AX11, N06AX16, N06AX17, N06AX21, and N06AX26 in oral formulation<br>or<br>drug code[a]: 1179034	Covariate
Antipsychotic	WHO-ATC code: N05AA01, N05AA02, N05AB02, N05AB03, N05AB04, N05AC01, N05AD01, N05AD05, N05AD06, N05AD08, N05AE01, N05AE05, N05AG02, N05AH02, N05AH03, N05AH04, N05AH05, N05AL01, N05AL02, N05AL03, N05AN01, N05AX08, N05AX10, N05AX11, N05AX12, N05AX13, and N05AX16 in oral and injectable formulations<br>or<br>Drug code[a]: 1179015, 1179026, 1179030, 1179036, 1179043, 1179048, 1179100, 1179101, 1179405, 1179700	Covariate
Neuroleptic	WHO-ATC code: N02BF02, N02BF03, and N02BG11 in oral formulation	Covariate
Antiepileptic	WHO-ATC code: N03AA02, N03AA03, N03AB01, N03AB02, N03AB52, N03AC02, N03AD01, N03AE01, N03AF01, N03AF03, N03AG01, N03AG03, N03AG04, N03AX03, N03AX09, N03AX11, N03AX12, N03AX13, N03AX14, N03AX15, N03AX17, N03AX18, N03AX22, and N03AX26 in oral formulation<br>or<br>drug code[a]: 1139700	Covariate

eTable 1. Definitions for variables (Continued)

Variables	Definitions	Purposes
Drugs		
Laxative	WHO-ATC code: A06AB02, A06AB05, A06AB06, A06AB08, A06AB56, A06AB57, A06AC06, A06AC08, A06AD04, A06AD10, A06AD11, A06AD12, A06AD65, A06AG04, A06AH05, A06AX02, A06AX03, A06AX04, and A06AX09 in oral formulation<br>or<br>Drug code[a]: 2359008	Covariate
Traditional herbal medicine	WHO-ATC code: A06AB06, C01EB04, G02CX04, M02AB01, N05CM09, R03CA02, and R05CA04 in oral formulation<br>or<br>drug code[a]: 510-, 520-, and 590-	Covariate
Start year of follow-up	The year of the index date	Covariate

[a] Procedure, drug, and claims codes were local ones in Japan.

Abbreviations: ACE, angiotensin-converting enzyme; ADP, adenosine diphosphate receptor antagonist; ARB, angiotensin II receptor blocker; ARNI, angiotensin receptor–neprilysin inhibitor; BZD, benzodiazepine; CCB, calcium channel blocker; DCI, drug comorbidity index; DOAC, direct oral anticoagulant; DPP-4, dipeptidyl peptidase-4; GLP-1, glucagon-like peptide-1 receptor agonist; ICD-10, international classification of diseases, 10th revision; MRA, mineralocorticoid receptor antagonist; NSAID, non-steroidal anti-inflammatory drug; NVAF, nonvalvular atrial fibrillation; PPI, proton pump inhibitor; SD, standard deviation; SGLT-2, sodium-glucose cotransporter 2; SU, sulfonylurea; VKA, vitamin K antagonist; WHO-ATC, world health organization-anatomical therapeutic chemical.

© 2026 Iketani R et al. *JAMA Network Open*.

eTable 2. Definitions for inclusion and exclusion criteria

	Definition
**Inclusion criteria**	
Continuous use of pharmacies for the treatment of chronic diseases	Visiting a pharmacy at least twice to treat primary diseases, with drugs prescribed for the diseases within 1 year before the cohort entry date, requiring a 1-year look-back period
Aged ≥ 75 years	Being aged 75 years or older at the cohort entry date
**Exclusion criteria**	
History of home medical care	Receiving home medical care within 1 year before the cohort entry and index date
History of dialysis	Receiving dialysis within 1 year before the cohort entry and index date
History of tumors	Having tumor within 1 year before the cohort entry and index date
History of Alzheimer's disease	Having Alzheimer's disease within 1 year before the cohort entry and index date
Prior FPS use	Claimed for "family pharmacist consultation fee" within 1 year before the cohort entry date
Outcome events on the cohort entry date	Hospitalized or died on the cohort entry date
Unavailable death records	Death records being unavailable on the cohort entry date
Comprehensive claims for dispensing fees	Claimed for "comprehensive management fee for family pharmacist (claims code: 440004510)" within 1 year before the cohort entry and index date

Abbreviation: FPS, family pharmacist system

© 2026 Iketani R et al. *JAMA Network Open*.

eTable 3. Incidence and risk of supportive secondary end points after matching

Group	No. of patients	No. of events	Person-years	Incidence rate (95% CI), per 1000 person-years	HR (95% CI)
**Supportive secondary end point**					
Death or unplanned hospitalization[a] from any cause:					
Users	22557	3340	32607.7	102.4 (99.0-106.0)	0.97 (0.92-1.01)
Nonusers	22557	3420	32228.5	106.1 (102.6-109.7)	1 [Reference]
Unplanned hospitalization[a] from any cause:					
Users	22557	2872	32607.7	88.1 (84.9-91.4)	0.98 (0.93-1.03)
Nonusers	22557	2907	32228.5	90.2 (87.0-93.5)	1 [Reference]

[a] Unplanned hospitalization was defined as claims of “additional fee for emergency medical care management (procedure code: A205 AND A999)” or hospitalization fee claim (procedure code: A300, A301-1, A301-2, and A301-3) on the initial day of hospitalization.

Abbreviations: CI, confidence interval; HR, hazard ratio.

© 2026 Iketani R et al. *JAMA Network Open*.

eTable 4. Discrete-time survival analysis with the inverse probability of censoring weighting after matching (1 year)

Group	No. of patients	After 1 year % (95% CI)	RR (95% CI)	ARD (%) (95% CI)	NNT
**Intention-to-treat**					
Death or hospitalization from any cause:					
Users	22557	19.3 (18.7 to 19.9)	0.99 (0.95 to 1.03)	-0.3 (-1.1 to 0.5)	356
Nonusers	22557	19.6 (19.1 to 20.1)	1 [Reference]	0 [Reference]	NA
Death from any cause:					
Users	22557	3.0 (2.7 to 3.3)	0.94 (0.82 to 1.05)	-0.2 (-0.6 to 0.1)	479
Nonusers	22557	3.2 (3.0 to 3.4)	1 [Reference]	0 [Reference]	NA
Hospitalization from any cause:					
Users	22557	18.8 (18.2 to 19.4)	0.99 (0.95 to 1.04)	-0.1 (-0.9 to 0.7)	880
Nonusers	22557	18.9 (18.4 to 19.4)	1 [Reference]	0 [Reference]	NA
Prescription change:					
Users	22557	14.2 (13.7 to 14.8)	1.61 (1.53 to 1.71)	5.4 (4.8 to 6.1)	19
Nonusers	22557	8.8 (8.4 to 9.2)	1 [Reference]	0 [Reference]	NA
**Per-protocol**					
Death or hospitalization from any cause:					
Users	22557	19.7 (19.0 to 20.4)	1.00 (0.96 to 1.05)	0.0 (-0.9 to 0.9)	NA
Nonusers	22557	19.6 (19.1 to 20.2)	1 [Reference]	0 [Reference]	NA
Death from any cause:					
Users	22557	3.4 (3.1 to 3.7)	1.01 (0.90 to 1.14)	0.0 (-0.4 to 0.5)	NA
Nonusers	22557	3.4 (3.1 to 3.6)	1 [Reference]	0 [Reference]	NA

© 2026 Iketani R et al. *JAMA Network Open*.

eTable 4. Discrete-time survival analysis with the inverse probability of censoring weighting after matching (1 year) (Continued)

Group	No. of patients	After 1 year			
		% (95% CI)	RR (95% CI)	ARD (%) (95% CI)	NNT
**Per-protocol**					
Hospitalization from any cause:					
Users	22557	19.1 (18.4 to 19.7)	1.01 (0.96 to 1.06)	0.2 (-0.7 to 1.1)	NA
Nonusers	22557	18.9 (18.4 to 19.4)	1 [Reference]	0 [Reference]	NA
Prescription change:					
Users	22557	14.3 (13.8 to 14.9)	1.60 (1.52 to 1.70)	5.3 (4.7 to 6.0)	19
Nonusers	22557	9.0 (8.5 to 9.4)	1 [Reference]	0 [Reference]	NA

Abbreviations: ARD, absolute risk difference; CI, confidence interval; NA, not applicable; NNT, number needed to treat; RR, risk ratio

© 2026 Iketani R et al. *JAMA Network Open*.

eTable 5. Discrete-time survival analysis with the inverse probability of censoring weighting after matching (2 years)

Group	No. of patients	After 2 years % (95% CI)	RR (95% CI)	ARD (%) (95% CI)	NNT
**Intention-to-treat**					
Death or hospitalization from any cause:					
Users	22557	34.3 (33.4 to 35.3)	0.99 (0.95 to 1.03)	-0.4 (-1.8 to 1.0)	281
Nonusers	22557	34.7 (33.6 to 35.9)	1 [Reference]	0 [Reference]	NA
Death from any cause:					
Users	22557	7.4 (7.0 to 7.9)	0.87 (0.77 to 0.98)	-1.2 (-2.1 to -0.2)	86
Nonusers	22557	8.6 (7.8 to 9.6)	1 [Reference]	0 [Reference]	NA
Hospitalization from any cause:					
Users	22557	33.3 (32.4 to 34.2)	1.00 (0.97 to 1.04)	0.1 (-1.1 to 1.3)	NA
Nonusers	22557	33.2 (32.3 to 34.1)	1 [Reference]	0 [Reference]	NA
Prescription change:					
Users	22557	23.0 (22.3 to 23.8)	1.51 (1.42 to 1.59)	7.7 (6.7 to 8.8)	13
Nonusers	22557	15.3 (14.6 to 16.1)	1 [Reference]	0 [Reference]	NA
**Per-protocol**					
Death or hospitalization from any cause:					
Users	22557	34.8 (33.7 to 35.9)	1.00 (0.96 to 1.05)	0.0 (-1.4 to 1.6)	NA
Nonusers	22557	34.8 (33.5 to 36.1)	1 [Reference]	0 [Reference]	NA
Death from any cause:					
Users	22557	8.2 (7.6 to 8.8)	0.89 (0.79 to 1.02)	-1.1 (-2.2 to 0.1)	95
Nonusers	22557	9.2 (8.3 to 10.4)	1 [Reference]	0 [Reference]	NA

© 2026 Iketani R et al. *JAMA Network Open*.

eTable 5. Discrete-time survival analysis with the inverse probability of censoring weighting after matching (2 years) (Continued)

Group	No. of patients	After 2 years			
		% (95% CI)	RR (95% CI)	ARD (%) (95% CI)	NNT
**Per-protocol**					
Hospitalization from any cause:					
Users	22557	33.8 (32.6 to 34.8)	1.02 (0.97 to 1.06)	0.6 (-1.0 to 2.0)	NA
Nonusers	22557	33.2 (32.2 to 34.2)	1 [Reference]	0 [Reference]	NA
Prescription change:					
Users	22557	22.4 (21.5 to 23.4)	1.45 (1.36 to 1.57)	6.9 (5.6 to 8.3)	14
Nonusers	22557	15.5 (14.7 to 16.3)	1 [Reference]	0 [Reference]	NA

Abbreviations: ARD, absolute risk difference; CI, confidence interval; NA, not applicable; NNT, number needed to treat; RR, risk ratio

© 2026 Iketani R et al. *JAMA Network Open*.

eTable 6. Baseline characteristics before and after matching

Characteristic	Patients, No. (%)					
	After matching			Before matching[a]		
	Users (n = 22557)	Nonusers (n = 22557)	SMD	Users (n = 22563)	Nonusers (n = 22563)	SMD
Age, mean (SD), y	82.8 (5.0)	82.8 (5.4)	0.00	82.8 (5.0)	82.2 (5.1)	0.12
Sex						
Female	16159 (71.6)	16095 (71.4)	0.01	16163 (71.6)	15567 (69.0)	0.06
Male	6398 (28.4)	6462 (28.6)	0.01	6400 (28.4)	6996 (31.0)	0.06
DCI, mean (SD)	1.4 (1.2)	1.5 (1.3)	0.02	1.4 (1.2)	1.3 (1.2)	0.12
Number of hospitalizations						
0	19121 (84.8)	19012 (84.3)	0.01	19123 (84.8)	19973 (88.5)	0.11
1	2647 (11.7)	2733 (12.1)	0.01	2649 (11.7)	2092 (9.3)	0.08
2	628 (2.8)	638 (2.8)	0.00	629 (2.8)	420 (1.9)	0.06
≥ 3	161 (0.7)	174 (0.8)	0.01	162 (0.7)	78 (0.3)	0.05
Number of physician visits						
≤ 12	5988 (26.5)	5948 (26.4)	0.00	5991 (26.6)	4910 (21.8)	0.11
13-24	8358 (37.1)	8344 (37.0)	0.00	8360 (37.1)	8255 (36.6)	0.01
≥ 25	8211 (36.4)	8265 (36.6)	0.00	8212 (36.4)	9398 (41.7)	0.11
Number of medical facilities used						
1	4273 (18.9)	4240 (18.8)	0.00	4276 (19.0)	3967 (17.6)	0.04
2	6071 (26.9)	6066 (26.9)	0.00	6072 (26.9)	5795 (25.7)	0.03
≥ 3	12213 (54.1)	12251 (54.3)	0.00	12215 (54.1)	12801 (56.7)	0.05
Dispensing in the hospital	15825 (70.2)	15893 (70.5)	0.01	15829 (70.2)	16069 (71.2)	0.02

© 2026 Iketani R et al. *JAMA Network Open*.

eTable 6. Baseline characteristics before and after matching (Continued)

Characteristic	Patients, No. (%)					
	After matching			Before matching[a]		
	Users (n = 22557)	Nonusers (n = 22557)	SMD	Users (n = 22563)	Nonusers (n = 22563)	SMD
Surgery	3304 (14.6)	3281 (14.5)	0.00	3306 (14.7)	2991 (13.3)	0.04
Number of drug types taken						
≤ 5	1834 (8.1)	1805 (8.0)	0.00	1835 (8.1)	2287 (10.1)	0.07
6-12	7756 (34.4)	7678 (34.0)	0.01	7757 (34.4)	7801 (34.6)	0.00
≥ 13	12967 (57.5)	13074 (58.0)	0.01	12971 (57.5)	12475 (55.3)	0.04
Primary disease						
Hypertension	20405 (90.5)	20414 (90.5)	0.00	20410 (90.5)	19970 (88.5)	0.06
Type 2 diabetes	10430 (46.2)	10518 (46.6)	0.01	10435 (46.2)	9788 (43.4)	0.06
Hyperlipidemia	10889 (48.3)	10940 (48.5)	0.00	10892 (48.3)	10651 (47.2)	0.02
Heart failure	8190 (36.3)	8153 (36.1)	0.00	8195 (36.3)	6616 (29.3)	0.15
Angina	6018 (26.7)	5890 (26.1)	0.01	6021 (26.7)	4981 (22.1)	0.11
NVAF	3156 (14.0)	3133 (13.9)	0.00	3161 (14.0)	2469 (10.9)	0.09
Arrhythmia other than NVAF	4030 (17.9)	3844 (17.0)	0.02	4033 (17.9)	3556 (15.8)	0.06
Comorbidity						
Vascular disease	4780 (21.2)	4715 (20.9)	0.01	4782 (21.2)	4394 (19.5)	0.04
Stroke	4042 (17.9)	4083 (18.1)	0.00	4045 (17.9)	3399 (15.1)	0.08
Valvular disease	4265 (18.9)	4156 (18.4)	0.01	4270 (18.9)	3451 (15.3)	0.10
Venous thromboembolism	800 (3.5)	745 (3.3)	0.01	802 (3.6)	570 (2.5)	0.06
Other embolisms	938 (4.2)	910 (4.0)	0.01	939 (4.2)	752 (3.3)	0.04

© 2026 Iketani R et al. *JAMA Network Open*.

eTable 6. Baseline characteristics before and after matching (Continued)

	Patients, No. (%)					
	After matching			Before matching[a]		
Characteristic	Users (n = 22557)	Nonusers (n = 22557)	SMD	Users (n = 22563)	Nonusers (n = 22563)	SMD
Comorbidity						
Pacemaker placement	661 (2.9)	538 (2.4)	0.03	665 (2.9)	291 (1.3)	0.12
Gastrointestinal hemorrhage	291 (1.3)	302 (1.3)	0.00	291 (1.3)	257 (1.1)	0.01
Kidney disease	3270 (14.5)	3336 (14.8)	0.01	3275 (14.5)	2805 (12.4)	0.06
Liver disease	3599 (16.0)	3713 (16.5)	0.01	3600 (16.0)	3679 (16.3)	0.01
Drug						
CCB with mainly vascular effects	15141 (67.1)	15227 (67.5)	0.01	15144 (67.1)	14873 (65.9)	0.03
CCB with direct cardiac effects	890 (3.9)	890 (3.9)	0.00	891 (3.9)	760 (3.4)	0.03
Other CCB	101 (0.4)	102 (0.5)	0.00	101 (0.4)	73 (0.3)	0.02
ACE	1426 (6.3)	1425 (6.3)	0.00	1426 (6.3)	1245 (5.5)	0.03
ARB	12362 (54.8)	12352 (54.8)	0.00	12364 (54.8)	11874 (52.6)	0.04
ARNI	197 (0.9)	181 (0.8)	0.01	197 (0.9)	106 (0.5)	0.05
Non-selective beta blocker	130 (0.6)	150 (0.7)	0.01	130 (0.6)	122 (0.5)	0.00
Selective beta blocker	3130 (13.9)	2986 (13.2)	0.02	3131 (13.9)	2591 (11.5)	0.07
Alpha and beta blocker	1851 (8.2)	1809 (8.0)	0.01	1854 (8.2)	1445 (6.4)	0.07
Loop diuretic	3400 (15.1)	3407 (15.1)	0.00	3405 (15.1)	2396 (10.6)	0.13
Thiazide	1771 (7.9)	1837 (8.1)	0.01	1771 (7.8)	1580 (7.0)	0.03
MRA	1679 (7.4)	1693 (7.5)	0.00	1682 (7.5)	1175 (5.2)	0.09
Other diuretics	595 (2.6)	586 (2.6)	0.00	595 (2.6)	543 (2.4)	0.01

© 2026 Iketani R et al. *JAMA Network Open*.

eTable 6. Baseline characteristics before and after matching (Continued)

Characteristic	Patients, No. (%)					
	After matching			Before matching[a]		
	Users (n = 22557)	Nonusers (n = 22557)	SMD	Users (n = 22563)	Nonusers (n = 22563)	SMD
Drug						
Antiarrhythmic drug	938 (4.2)	910 (4.0)	0.01	939 (4.2)	710 (3.1)	0.05
Fibrate	876 (3.9)	840 (3.7)	0.01	877 (3.9)	912 (4.0)	0.01
Statin	11189 (49.6)	11109 (49.2)	0.01	11191 (49.6)	11058 (49.0)	0.01
Ezetimibe	1035 (4.6)	1015 (4.5)	0.00	1035 (4.6)	913 (4.0)	0.03
Other oral lipid-modifying agents	1398 (6.2)	1408 (6.2)	0.00	1398 (6.2)	1275 (5.7)	0.02
Other injectable lipid-modifying agents	14 (0.1)	9 (0.0)	0.01	14 (0.1)	6 (0.0)	0.02
Other heart failure drugs	761 (3.4)	740 (3.3)	0.01	765 (3.4)	467 (2.1)	0.08
VKA	795 (3.5)	756 (3.4)	0.01	797 (3.5)	572 (2.5)	0.06
DOAC	2280 (10.1)	2199 (9.7)	0.01	2283 (10.1)	1772 (7.9)	0.08
Low-dose aspirin	4031 (17.9)	3995 (17.7)	0.00	4032 (17.9)	3492 (15.5)	0.06
Nitroglycerin	866 (3.8)	830 (3.7)	0.01	867 (3.8)	725 (3.2)	0.03
ADP inhibitors other than ticlopidine	1922 (8.5)	1874 (8.3)	0.01	1922 (8.5)	1546 (6.9)	0.06
Ticlopidine	148 (0.7)	127 (0.6)	0.01	148 (0.7)	114 (0.5)	0.02
SU	1286 (5.7)	1245 (5.5)	0.01	1286 (5.7)	1154 (5.1)	0.03
DPP-4 inhibitor	3969 (17.6)	3966 (17.6)	0.00	3971 (17.6)	3693 (16.4)	0.03
SGLT-2 inhibitor	1160 (5.1)	1155 (5.1)	0.00	1163 (5.2)	937 (4.2)	0.05
GLP-1 agonist	231 (1.0)	236 (1.0)	0.00	232 (1.0)	161 (0.7)	0.03
Insulin	943 (4.2)	971 (4.3)	0.01	946 (4.2)	722 (3.2)	0.05

© 2026 Iketani R et al. *JAMA Network Open*.

eTable 6. Baseline characteristics before and after matching (Continued)

Characteristic	Patients, No. (%)					
	After matching			Before matching[a]		
	Users (n = 22557)	Nonusers (n = 22557)	SMD	Users (n = 22563)	Nonusers (n = 22563)	SMD
Drug						
Biguanide	1710 (7.6)	1803 (8.0)	0.02	1710 (7.6)	1580 (7.0)	0.02
Other hypoglycemic drugs	1864 (8.3)	1917 (8.5)	0.01	1866 (8.3)	1632 (7.2)	0.04
Stroke drug	137 (0.6)	127 (0.6)	0.01	137 (0.6)	101 (0.4)	0.02
Subarachnoid hemorrhage drug	67 (0.3)	70 (0.3)	0.00	67 (0.3)	38 (0.2)	0.03
NSAIDs	16401 (72.7)	16294 (72.2)	0.01	16405 (72.7)	16332 (72.4)	0.01
Acetaminophen	6598 (29.3)	6690 (29.7)	0.01	6601 (29.3)	6112 (27.1)	0.05
Opioid	1457 (6.5)	1540 (6.8)	0.01	1457 (6.5)	1245 (5.5)	0.04
Steroid	7932 (35.2)	7973 (35.3)	0.00	7932 (35.2)	7868 (34.9)	0.01
BZD	5696 (25.3)	5736 (25.4)	0.00	5699 (25.3)	5251 (23.3)	0.05
Non-BZD	2813 (12.5)	2876 (12.7)	0.01	2816 (12.5)	2622 (11.6)	0.03
Orexin receptor blocker	1329 (5.9)	1355 (6.0)	0.00	1330 (5.9)	1048 (4.6)	0.06
H2 blocker	2488 (11.0)	2597 (11.5)	0.02	2488 (11.0)	2439 (10.8)	0.01
PPI	8904 (39.5)	8853 (39.2)	0.00	8908 (39.5)	7396 (32.8)	0.14
Antibiotic	7621 (33.8)	7649 (33.9)	0.00	7624 (33.8)	7904 (35.0)	0.03
Antidepressant	1452 (6.4)	1412 (6.3)	0.01	1453 (6.4)	1334 (5.9)	0.02
Antipsychotic	952 (4.2)	1049 (4.7)	0.02	954 (4.2)	817 (3.6)	0.03
Neuroleptic	2360 (10.5)	2381 (10.6)	0.00	2360 (10.5)	2168 (9.6)	0.03
Antiepileptic	545 (2.4)	603 (2.7)	0.02	545 (2.4)	449 (2.0)	0.03

© 2026 Iketani R et al. *JAMA Network Open*.

eTable 6. Baseline characteristics before and after matching (Continued)

	Patients, No. (%)					
	After matching			Before matching[a]		
Characteristic	Users (n = 22557)	Nonusers (n = 22557)	SMD	Users (n = 22563)	Nonusers (n = 22563)	SMD
Drug						
Lexative	5153 (22.8)	5337 (23.7)	0.02	5157 (22.9)	4424 (19.6)	0.08
Traditional herbal medicine	6288 (27.9)	6203 (27.5)	0.01	6290 (27.9)	6178 (27.4)	0.01
Start year of follow-up						
2017	2593 (11.5)	2596 (11.5)	0.00	2595 (11.5)	1482 (6.6)	0.17
2018	1331 (5.9)	1331 (5.9)	0.00	1331 (5.9)	1633 (7.2)	0.05
2019	2045 (9.1)	2023 (9.0)	0.00	2045 (9.1)	3985 (17.7)	0.25
2020	4156 (18.4)	4142 (18.4)	0.00	4157 (18.4)	4340 (19.2)	0.02
2021	5915 (26.2)	5841 (25.9)	0.00	5916 (26.2)	4972 (22.0)	0.10
2022	4950 (21.9)	5043 (22.4)	0.00	4951 (21.9)	4661 (20.7)	0.03
2023	1560 (6.9)	1575 (7.0)	0.00	1561 (6.9)	1485 (6.6)	0.01
2024	7 (0.0)	6 (0.0)	0.01	7 (0.0)	5 (0.0)	0.01

[a] Nonusers before matching were randomly selected to ensure that the numbers of users and nonusers were balanced within each time-based exposure set.

Abbreviations: ACE, angiotensin-converting enzyme; ADP, adenosine diphosphate receptor antagonist; ARB, angiotensin II receptor blocker; ARNI, angiotensin receptor–neprilysin inhibitor; BZD, benzodiazepine; CCB, calcium channel blocker; DCI, drug comorbidity index; DOAC, direct oral anticoagulant; DPP-4, dipeptidyl peptidase-4; GLP-1, glucagon-like peptide-1 receptor agonist; MRA, mineralocorticoid receptor antagonist; NSAID, non-steroidal anti-inflammatory drug; NVAF, nonvalvular atrial fibrillation; PPI, proton pump inhibitor; SD, standard deviation; SGLT-2, sodium-glucose cotransporter 2; SU, sulfonylurea; SMD, standardized mean difference; and VKA, vitamin K antagonist.

© 2026 Iketani R et al. *JAMA Network Open*.

eTable 7. Incidence and risk of outcomes before matching

Group	No. of patients	No. of events	Person-years	Incidence rate (95% CI), /1000 person-years	HR (95% CI)
**Primary end point**					
Death or hospitalization from any cause:					
Users	22563	6354	29375.1	216.3 (211.0-221.7)	1.13 (1.10-1.18)
Nonusers[a]	22563	5760	30250.8	190.4 (185.6-195.4)	1 [Reference]
**Secondary end point**					
Death from any cause:					
Users	22563	1175	34681.2	33.9 (32.0-35.9)	1.15 (1.06-1.25)
Nonusers[a]	22563	1040	35177.5	29.6 (27.8-31.4)	1 [Reference]
Hospitalization from any cause:					
Users	22563	6184	29375.1	210.5 (205.3-215.8)	1.14 (1.10-1.18)
Nonusers[a]	22563	5583	30250.8	184.6 (179.8-189.5)	1 [Reference]
Prescription change:					
Users	22563	4398	30258.9	145.3 (141.1-149.7)	1.67 (1.60-1.76)
Nonusers[a]	22563	2796	32469.3	86.1 (83.0-89.4)	1 [Reference]

[a] Nonusers before matching were randomly selected to ensure that the numbers of users and nonusers were balanced within each time-based exposure set.

Abbreviations: CI, confidence interval; HR, hazard ratio.

© 2026 Iketani R et al. *JAMA Network Open*.

eTable 8. Sensitivity analysis of changing the matching ratio to 1:2 (user: nonuser)

Group	No. of patients	No. of events	Person-years	Incidence rate (95% CI), per 1000 person-years	HR (95% CI)
**Primary end point**					
Death or hospitalization from any cause:					
Users	22557	6351	29371.1	216.2 (211.0-221.6)	0.99 (0.96-1.03)
Nonusers	45114	12638	58108.3	217.5 (213.7-221.3)	1 [Reference]
**Secondary end point**					
Death from any cause:					
Users	22557	1174	34673.3	33.9 (32.0-35.9)	0.93 (0.87-0.996)
Nonusers	45114	2497	68623.0	36.4 (35.0-37.8)	1 [Reference]
Hospitalization from any cause:					
Users	22557	6181	29371.1	210.4 (205.3-215.8)	1.00 (0.97-1.03)
Nonusers	45114	12216	58108.3	210.2 (206.5-214.0)	1 [Reference]
Prescription change:					
Users	22557	4397	30251.1	145.4 (141.1-149.7)	1.60 (1.54-1.67)
Nonusers	45114	5706	63113.7	90.4 (88.1-92.8)	1 [Reference]

Abbreviations: CI, confidence interval; HR, hazard ratio

© 2026 Iketani R et al. *JAMA Network Open*.

eTable 9. Sensitivity analysis of changing the definition of nonusers

Group	No. of patients	No. of events	Person-years	Incidence rate (95% CI), per 1000 person-years	HR (95% CI)
**Primary endpoint**					
Death or hospitalization from any cause:					
Users	22558	6351	29372.7	216.2 (211.0-221.6)	0.95 (0.92-0.98)
Nonusers	22558	6547	28747.0	227.7 (222.3-233.3)	1 [Reference]
**Secondary end point**					
Death from any cause:					
Users	22558	1175	34676.1	33.9 (32.0-35.9)	0.76 (0.71-0.82)
Nonusers	22558	1507	33987.8	44.3 (42.2-46.6)	1 [Reference]
Hospitalization from any cause:					
Users	22558	6181	29372.7	210.4 (205.3-215.7)	0.98 (0.95-1.02)
Nonusers	22558	6153	28747.0	214.0 (208.8-219.5)	1 [Reference]
Prescription change:					
Users	22558	4396	30254.6	145.3 (141.1-149.7)	1.64 (1.57-1.72)
Nonusers	22558	2762	31355.0	88.1 (84.9-91.4)	1 [Reference]

Abbreviations: CI, confidence interval; HR, hazard ratio.

© 2026 Iketani R et al. *JAMA Network Open*.

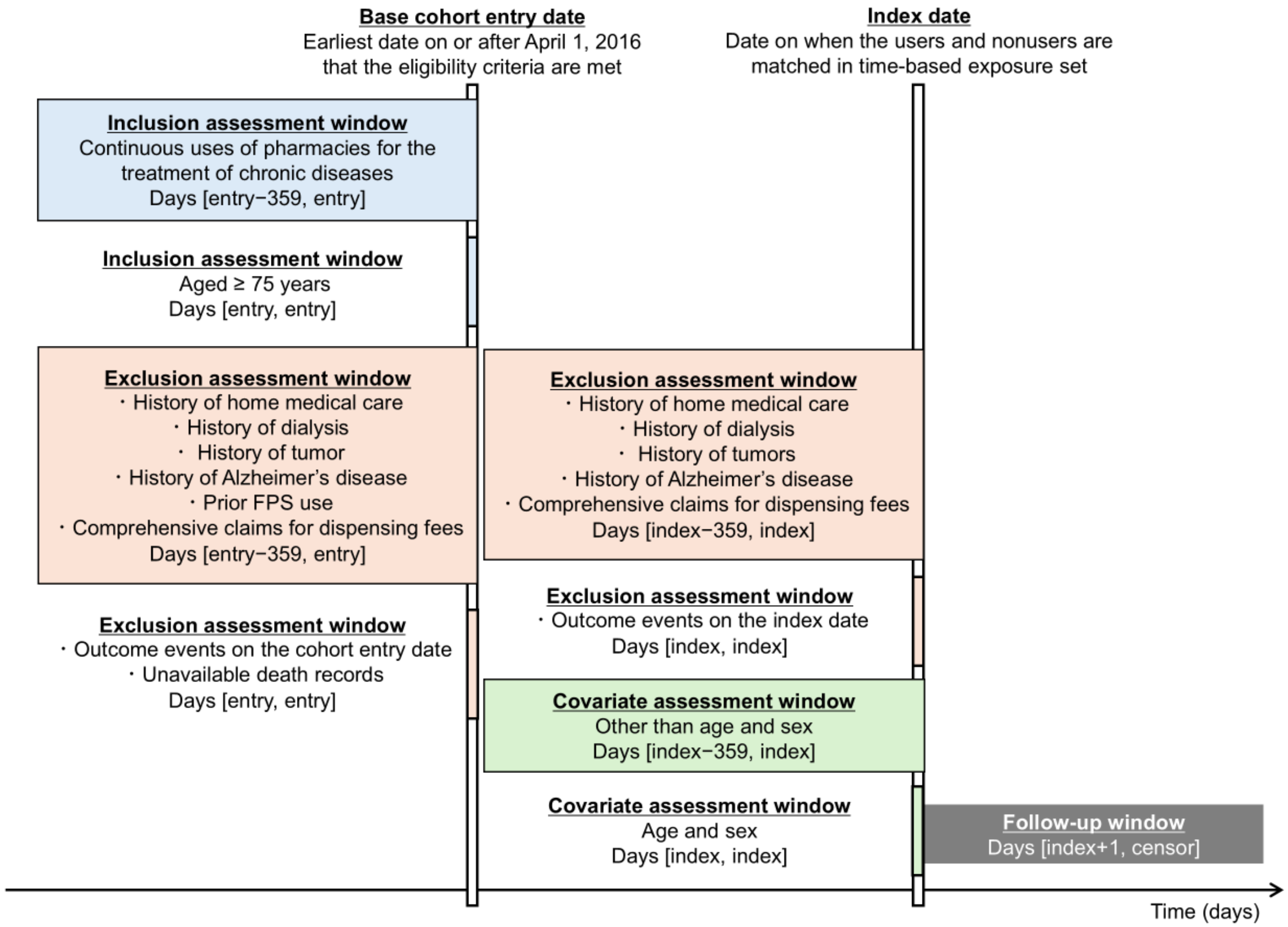

eFigure 1. Study design diagram

Abbreviation: FPS, family pharmacist system.

© 2026 Iketani R et al. *JAMA Network Open*.

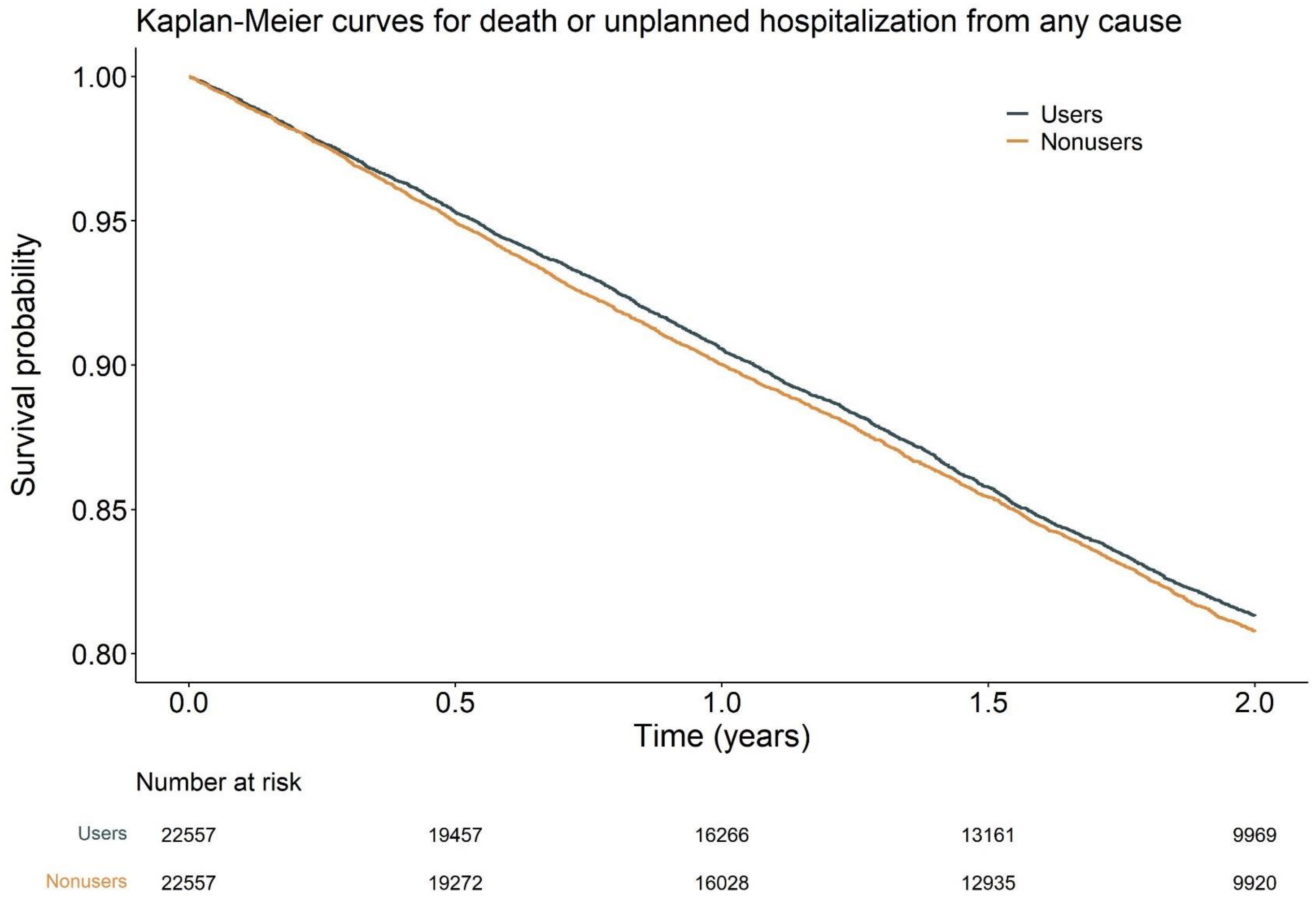

eFigure 2. Kaplan–Meier curves of death or unplanned hospitalization from any cause after matching

© 2026 Iketani R et al. *JAMA Network Open*.

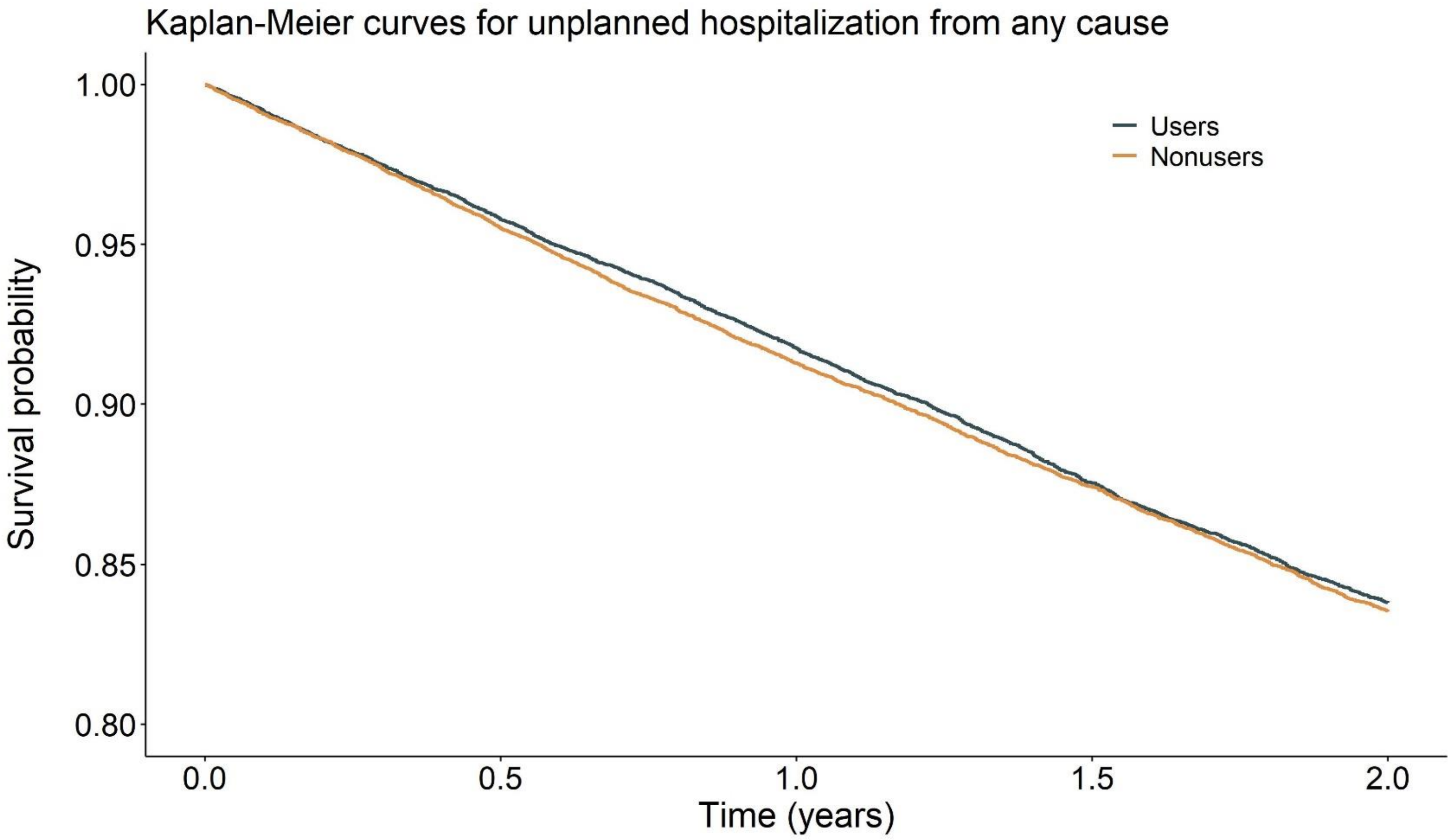

Number at risk

Users	22557	19457	16266	13161	9969
Nonusers	22557	19272	16028	12935	9920

eFigure 3. Kaplan–Meier curves of unplanned hospitalization from any cause after matching

© 2026 Iketani R et al. *JAMA Network Open*.

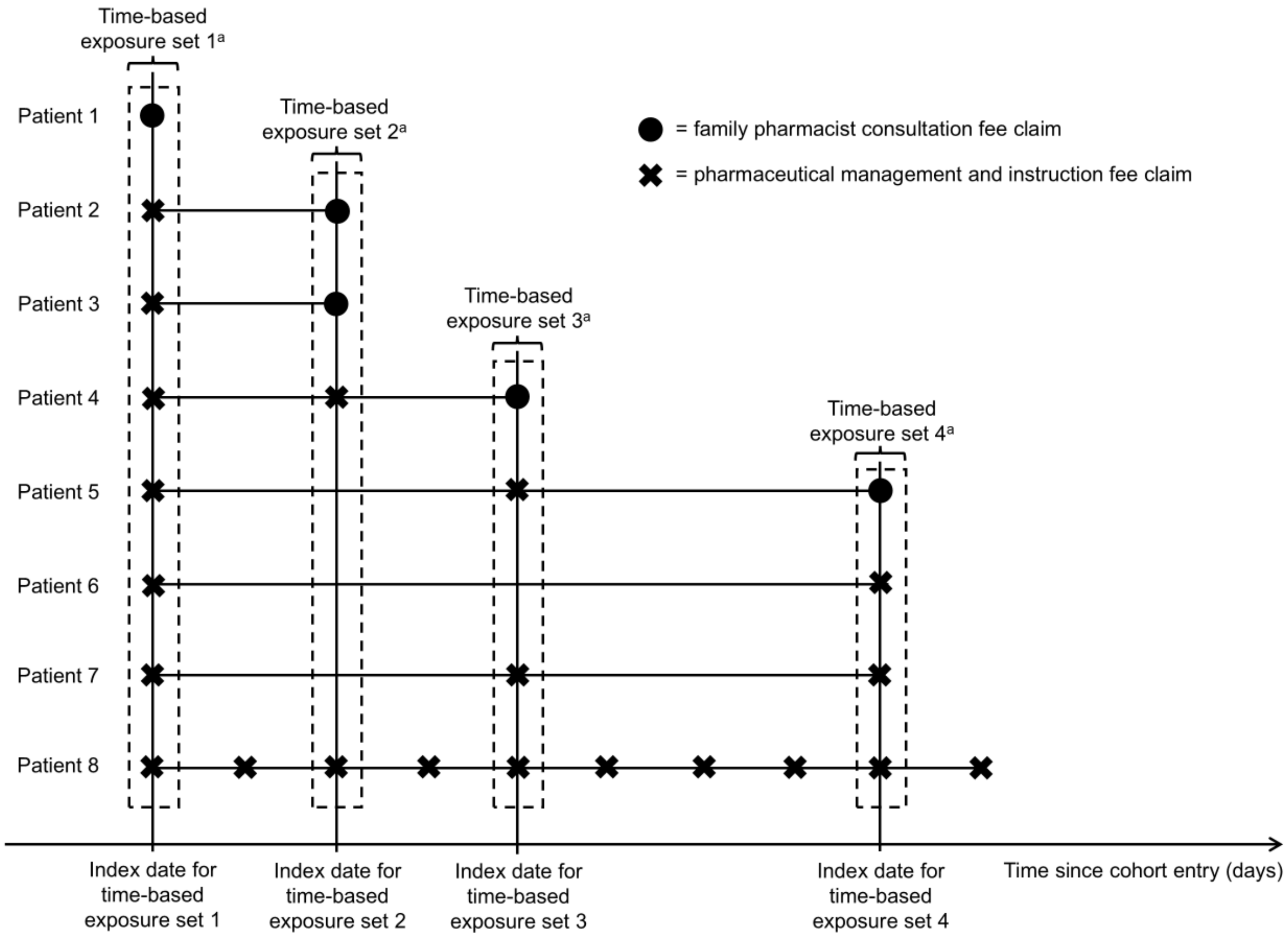

eFigure 4. Definition for time-based exposure set

[a] We created time-based exposure sets by pharmacy visit day from the cohort entry date, allowing no time intervals.

© 2026 Iketani R et al. *JAMA Network Open*.

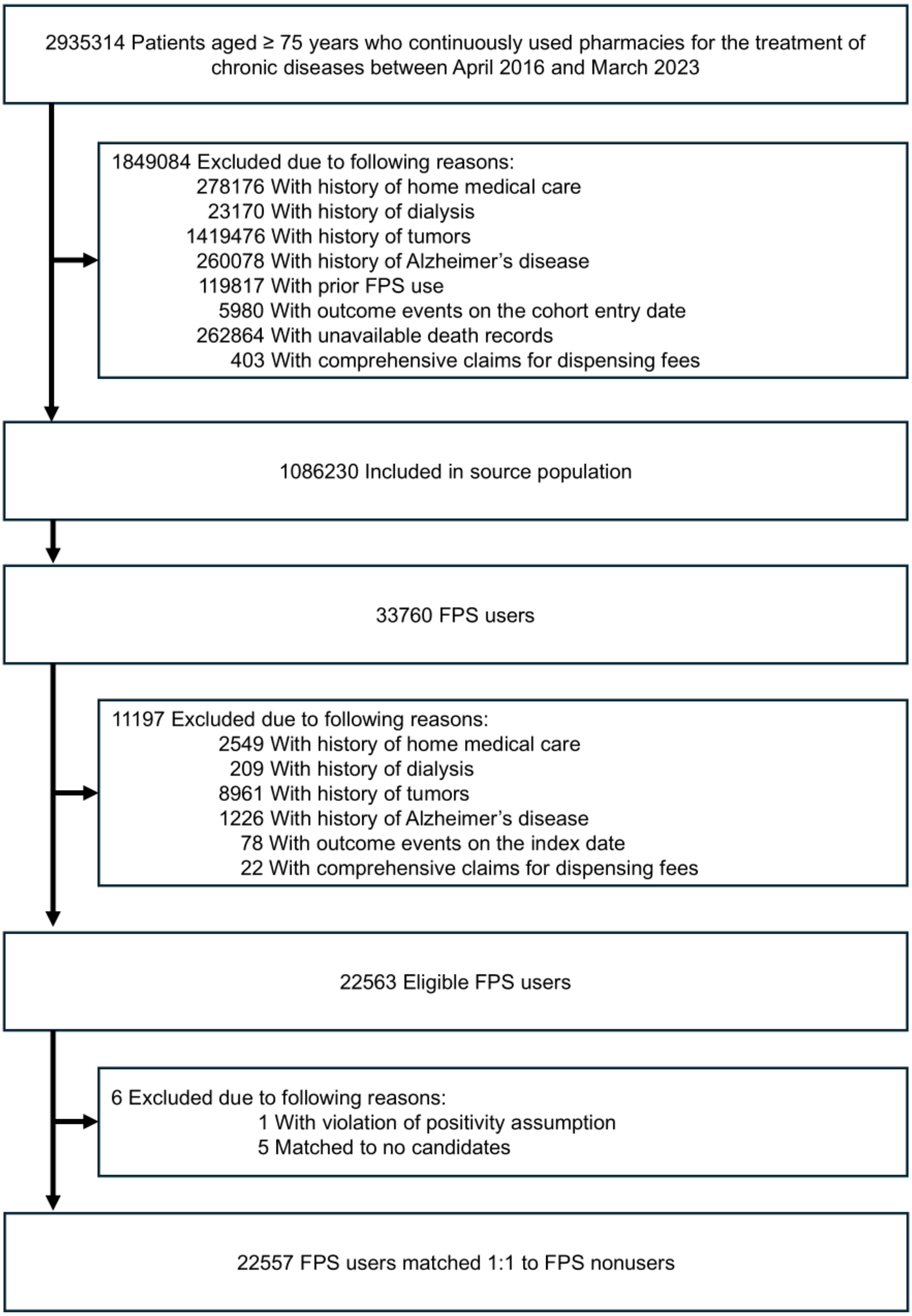

eFigure 5. Flowchart of study cohort selection

Abbreviation: FPS, family pharmacist system.

© 2026 Iketani R et al. *JAMA Network Open*.

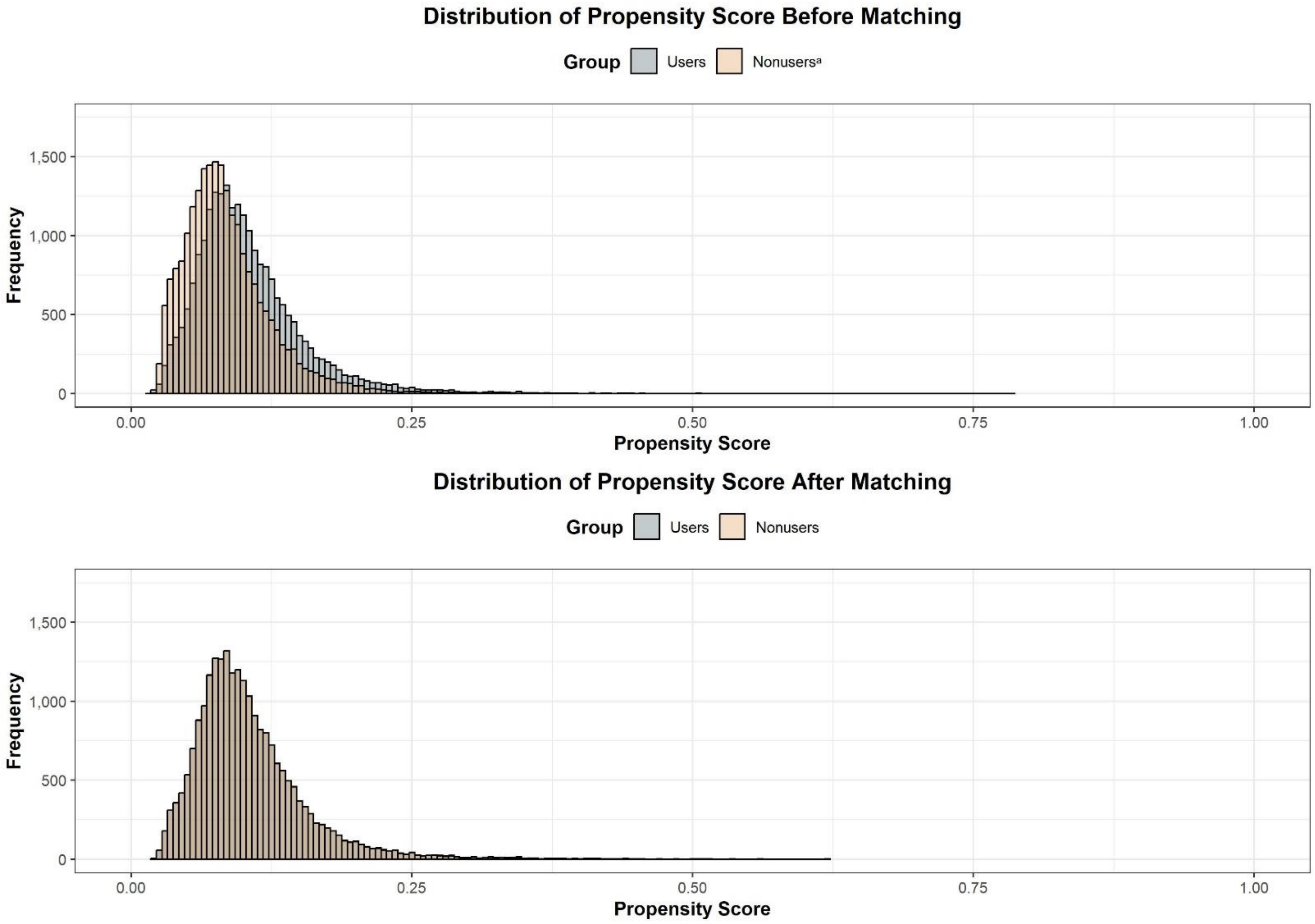

eFigure 6. Distribution of propensity score before and after matching

[a] Nonusers before matching were randomly selected to ensure that the numbers of users and nonusers were balanced within each time-based exposure set.

© 2026 Iketani R et al. *JAMA Network Open*.

Subgroup[a]	N	Rate of users (/1000 person-years)	Rate of nonusers (/1000 person-years)	RERI (95% CI)[b]	HR (95% CI)
Total	45114	33.9	37.0		0.91 (0.85 to 0.99)
Age				-0.26 (-0.63 to 0.12)	
< 85	29848	18.9	19.4		0.97 (0.85 to 1.11)
≥ 85	15254	64.7	68.1		0.95 (0.86 to 1.05)
Sex				-0.16 (-0.31 to -0.01)	
Male	12792	51.7	51.5		1.00 (0.89 to 1.13)
Female	32318	26.9	30.5		0.88 (0.79 to 0.98)
Hypertension				0.08 (-0.13 to 0.30)	
No	5972	30.5	37.5		0.81 (0.65 to 1.01)
Yes	39138	34.4	36.9		0.93 (0.86 to 1.01)
Type 2 diabetes				0.16 (-0.02 to 0.34)	
No	34338	32.2	35.5		0.91 (0.83 to 0.996)
Yes	10768	38.9	39.5		0.99 (0.84 to 1.15)
Hyperlipidemia				0.12 (-0.001 to 0.25)	
No	28220	38.2	42.7		0.89 (0.81 to 0.98)
Yes	16888	26.4	25.2		1.05 (0.90 to 1.22)
Heart failure				-0.48 (-0.82 to -0.15)	
No	34390	25.9	26.5		0.98 (0.88 to 1.09)
Yes	10714	60.2	72.1		0.83 (0.74 to 0.94)
Angina				-0.07 (-0.28 to 0.15)	
No	34614	31.2	33.9		0.92 (0.84 to 1.01)
Yes	10500	42.5	46.5		0.91 (0.79 to 1.06)
NVAF				-0.14 (-0.48 to 0.19)	
No	40016	31.3	33.9		0.92 (0.85 to 1.01)
Yes	5090	54.5	63.0		0.86 (0.72 to 1.04)
Arrhythmia other than NVAF				0.12 (-0.20 to 0.44)	
No	41728	33.2	36.5		0.91 (0.84 to 0.99)
Yes	3378	42.0	48.3		0.87 (0.67 to 1.12)

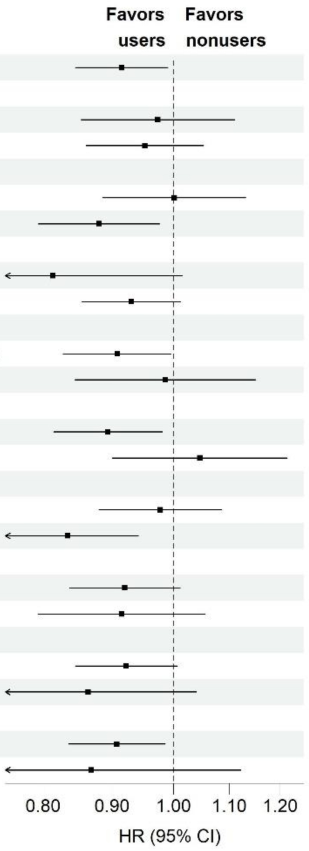

eFigure 7. Subgroup analysis for death from any cause

[a] Disease subgroups were defined on the basis of the presence of at least one prescription drug indicated for the corresponding condition.

[b] A positive relative excess risk due to interaction (RERI) value indicates an antagonistic additive interaction associated with age ≥ 85 years, female sex, or primary disease. Conversely, a negative RERI value suggests a synergistic additive interaction between these factors.

Abbreviations: CI, confidence interval; HR, hazard ratio; NVAF, nonvalvular atrial fibrillation; RERI, relative excess risk due to interaction.

© 2026 Iketani R et al. *JAMA Network Open*.

Subgroup[a]	N	Rate of users (/1000 person-years)	Rate of nonusers (/1000 person-years)	RERI (95% CI)[b]	HR (95% CI)
Total	45114	210.4	208.2		1.01 (0.98 to 1.05)
Age				-0.10 (-0.19 to -0.005)	
< 85	29848	188.5	182.2		1.03 (0.99 to 1.08)
≥ 85	15254	257.1	268.8		0.96 (0.91 to 1.01)
Sex				-0.07 (-0.14 to 0.004)	
Male	12792	258.9	247.4		1.05 (0.98 to 1.11)
Female	32318	192.2	190.2		1.01 (0.97 to 1.06)
Hypertension				0.08 (-0.02 to 0.18)	
No	5972	182.0	195.1		0.93 (0.84 to 1.03)
Yes	39138	214.8	209.3		1.03 (0.99 to 1.07)
Type 2 diabetes				0.03 (-0.07 to 0.12)	
No	34338	200.0	199.0		1.01 (0.96 to 1.05)
Yes	10768	245.2	250.0		0.98 (0.92 to 1.05)
Hyperlipidemia				0.02 (-0.05 to 0.09)	
No	28220	214.5	214.6		1.00 (0.96 to 1.04)
Yes	16888	203.6	191.8		1.06 (1.001 to 1.13)
Heart failure				-0.08 (-0.20 to 0.05)	
No	34390	179.0	172.2		1.04 (0.995 to 1.09)
Yes	10714	325.3	331.5		0.98 (0.92 to 1.04)
Angina				-0.02 (-0.13 to 0.09)	
No	34614	190.0	188.9		1.01 (0.96 to 1.05)
Yes	10500	282.5	285.8		0.99 (0.93 to 1.05)
NVAF				0.00 (-0.15 to 0.16)	
No	40016	196.4	193.9		1.01 (0.97 to 1.05)
Yes	5090	335.8	330.9		1.01 (0.93 to 1.11)
Arrhythmia other than NVAF				-0.11 (-0.29 to 0.06)	
No	41728	204.9	199.2		1.03 (0.99 to 1.07)
Yes	3378	285.1	308.4		0.93 (0.83 to 1.03)

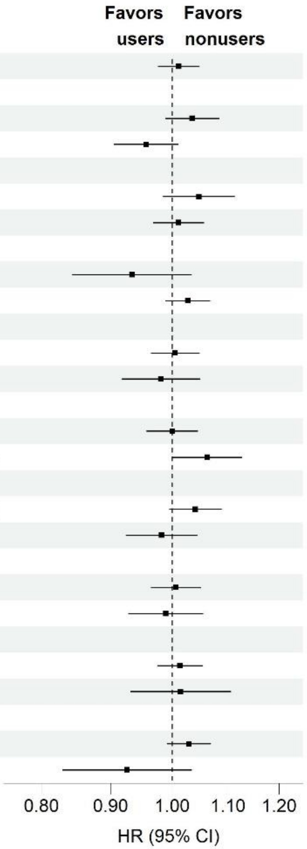

eFigure 8. Subgroup analysis for hospitalization from any cause

[a] Disease subgroups were defined on the basis of the presence of at least one prescription drug indicated for the corresponding condition.

[b] A positive relative excess risk due to interaction (RERI) value indicates an antagonistic additive interaction associated with age ≥ 85 years, female sex, or primary disease. Conversely, a negative RERI value suggests a synergistic additive interaction between these factors.

Abbreviations: CI, confidence interval; HR, hazard ratio; NVAF, nonvalvular atrial fibrillation; RERI, relative excess risk due to interaction.

© 2026 Iketani R et al. *JAMA Network Open*.

Subgroup[a]	N	Rate of users (/1000 person-years)	Rate of nonusers (/1000 person-years)	RERI (95% CI)[b]	HR (95% CI)
Total	45114	145.4	90.8		1.59 (1.52 to 1.67)
Age				0.07 (-0.05 to 0.20)	
< 85	29848	142.5	90.1		1.58 (1.49 to 1.67)
≥ 85	15254	151.3	96.2		1.56 (1.44 to 1.69)
Sex				0.01 (-0.12 to 0.15)	
Male	12792	138.8	84.7		1.63 (1.49 to 1.79)
Female	32318	147.9	92.6		1.59 (1.50 to 1.68)
Hypertension				0.03 (-0.15 to 0.21)	
No	5972	138.8	91.9		1.51 (1.33 to 1.71)
Yes	39138	146.3	91.9		1.59 (1.51 to 1.67)
Type 2 diabetes				-0.11 (-0.27 to 0.05)	
No	34338	141.1	84.8		1.66 (1.57 to 1.75)
Yes	10768	159.3	101.8		1.55 (1.41 to 1.70)
Hyperlipidemia				0.03 (-0.10 to 0.17)	
No	28220	138.6	85.4		1.62 (1.52 to 1.72)
Yes	16888	156.9	93.5		1.67 (1.55 to 1.80)
Heart failure				0.02 (-0.14 to 0.18)	
No	34390	139.3	84.5		1.64 (1.55 to 1.73)
Yes	10714	165.9	106.4		1.55 (1.41 to 1.70)
Angina				0.22 (0.05 to 0.38)	
No	34614	134.7	84.2		1.59 (1.51 to 1.69)
Yes	10500	181.7	111.7		1.62 (1.48 to 1.77)
NVAF				0.05 (-0.16 to 0.25)	
No	40016	143.3	89.1		1.60 (1.52 to 1.68)
Yes	5090	161.7	104.2		1.54 (1.35 to 1.76)
Arrhythmia other than NVAF				0.03 (-0.22 to 0.29)	
No	41728	143.8	89.7		1.60 (1.52 to 1.68)
Yes	3378	165.7	107.5		1.53 (1.30 to 1.80)

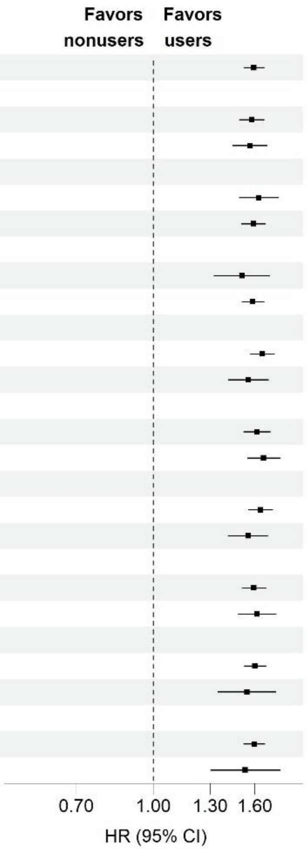

eFigure 9. Subgroup analysis for prescription changes

[a] Disease subgroups were defined on the basis of the presence of at least one prescription drug indicated for the corresponding condition.

[b] A positive relative excess risk due to interaction (RERI) value indicates a synergistic additive interaction associated with age ≥ 85 years, female sex, or primary disease. Conversely, a negative RERI value suggests an antagonistic additive interaction between these factors.

Abbreviations: CI, confidence interval; HR, hazard ratio; NVAF, nonvalvular atrial fibrillation; RERI, relative excess risk due to interaction.

© 2026 Iketani R et al. *JAMA Network Open*.
