# Supplementary material for: Family Pharmacist System for Patients With Chronic Cardiovascular or Endocrine Disease
Source: JAMA Netw Open. 2026 Feb 23;9(2):e2560398. doi: 10.1001/jamanetworkopen.2025.60398 (PMC12931473; doi:10.1001/jamanetworkopen.2025.60398)
Supplement: Supplement 2. — Data Sharing Statement [file jamanetwopen-e2560398-s002.pdf]

Data Sharing Statement

Iketani. Family Pharmacist System for Patients With Chronic Cardiovascular or Endocrine Disease. *JAMA Netw Open*. Published February 23, 2026.
doi:10.1001/jamanetworkopen.2025.60398

Data
Data available: No
